# Metric comparison of connectome-based lesion-symptom mapping in post-stroke aphasia

**DOI:** 10.1093/braincomms/fcae313

**Published:** 2024-09-12

**Authors:** Junhua Ding, Melissa Thye, Amelia J Edmondson-Stait, Jerzy P Szaflarski, Daniel Mirman

**Affiliations:** Department of Psychology, University of Edinburgh, Edinburgh EH8 9JZ, UK; Department of Psychology, University of Edinburgh, Edinburgh EH8 9JZ, UK; Department of Psychology, University of Edinburgh, Edinburgh EH8 9JZ, UK; Department of Neurology, University of Alabama at Birmingham, Birmingham, AL 35294, USA; Department of Psychology, University of Edinburgh, Edinburgh EH8 9JZ, UK

**Keywords:** stroke aphasia, multiverse analysis, lesion-symptom mapping, brain connectome, white matter tract

## Abstract

Connectome-based lesion-symptom mapping relates behavioural impairments to disruption of structural brain connectivity. Connectome-based lesion-symptom mapping can be based on different approaches (diffusion MRI versus lesion mask), network scales (whole brain versus regions of interest) and measure types (tract-based, parcel-based, or network-based metrics). We evaluated the similarity of different connectome-based lesion-symptom mapping processing choices and identified factors that influence the results using multiverse analysis—the strategy of conducting and displaying the results of all reasonable processing choices. Metrics derived from lesion masks and diffusion-weighted images were tested for association with Boston Naming Test and Token Test performance in a sample of 50 participants with aphasia following left hemispheric stroke. ‘Direct’ measures were derived from diffusion-weighted images. ‘Indirect’ measures were derived by overlaying lesion masks on a white matter atlas. Parcel-based connectomes were constructed for the whole brain and regions of interest (14 language-relevant parcels). Numerous tract-based and network-based metrics were calculated. There was a high discrepancy across processing approaches (diffusion-weighted images versus lesion masks), network scales (whole brain versus regions of interest) and metric types. Results indicate weak correlations and different connectome-based lesion-symptom mapping results across the processing choices. Substantial methodological work is needed to validate the various decision points that arise when conducting connectome-based lesion-symptom mapping analyses. Multiverse analysis is a useful strategy for evaluating the similarity across different processing choices in connectome-based lesion-symptom mapping.

## Teaser

Ding *et al*. report that in connectome-based lesion-symptom mapping on stroke patients, different metrics, imaging modality and network scales had a weak correlation and inconsistent results. More validation is needed for the various processing choices, and multiverse analysis is a useful strategy for evaluating their similarity.

## Graphical Abstract

**Graphical Abstract:**
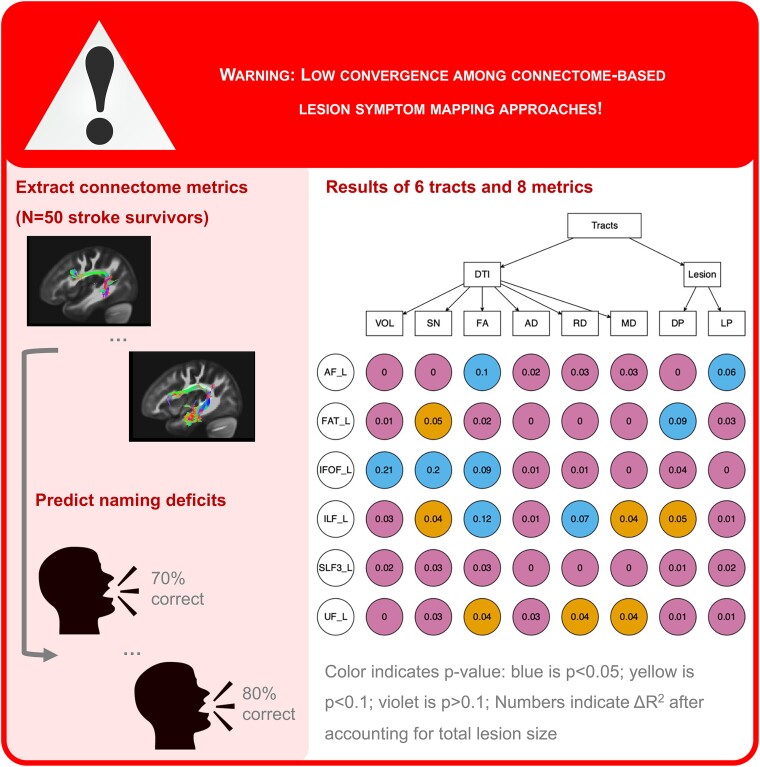

## Introduction

Lesion-symptom mapping is an important method for both clinical and theoretical questions about the neural basis of human behaviour and cognition and has been particularly applied to language processing.^1,2^ Voxel-based lesion-symptom mapping (VLSM)^3^ allows researchers to localize various deficits to the corresponding brain areas at a millimetre scale.^4-6^ However, focal brain damage cannot explain everything. Due to the network organization of the brain, damage to structural connections between brain regions—white matter tracts^7^—might exert an influence on symptoms as well. Even though information is not processed within white matter, these tracts serve as pathways for information transfer between critical regions, and this is likely to be particularly important for higher-level cognitive functions. For example, language processing relies on a complex network^8,9^ as well as communication with other cognitive systems such as semantic cognition.^10^ By considering the brain tissue as voxels, VLSM could be used to infer tract damage or disconnection, but this does not capture the fact that tracts are connections rather than processing units.

Connectome-based lesion-symptom mapping (CLSM) was developed as a complement to VLSM to evaluate how disruptions to the structural connections in the brain relate to behavioural symptoms, and the method has been quickly adopted.^11-13^ Using CLSM, researchers can examine associations between behaviour and disconnection of specific white matter tracts, construct brain parcel-based connectome networks or quantify the network structure of connectomes using graph theory metrics. Many published CLSM studies report effects of structural connectivity disruption beyond focal grey matter damage.^14-16^ However, CLSM analyses adopt different methods for constructing the connectomes, use different data sources and make different processing or statistical choices, and it is not clear what influence these possible decision points have on the results.

One significant source of variability is the approach used to generate the connectomes. Diffusion-weighted imaging (DWI) can be used to model how water molecules diffuse through tissue, and this information can be used to reconstruct areas of high diffusion (i.e. white matter tract bundles). DWI data are thus ideally suited for studying the structural connections in the brain and, as such, are often used for constructing connectomes, a so-called ‘direct’ approach.^17,18^ However, practical limitations, such as cost and clinical constraints, mean that often DWI data are not collected and only low-resolution structural MRI or CT images are available.^19,20^ As a result, researchers have adapted the CLSM approach to calculate connectome damage by overlapping lesion masks derived from structural images on a white matter atlas, the so-called ‘indirect’ approach.^21-24^ The measures generated from these indirect connectomes are intended to capture structural connectivity disruption beyond local lesion damage alone.^25-27^ Some studies have found a consistent pattern of results when comparing metrics such as fractional anisotropy (FA) and lesion load derived from DWI and lesion masks, respectively.^28,29^ A recent study found that functional connectomes derived directly from resting-state fMRI were superior for predicting multiple behavioural deficits compared to its indirect substitute derived from overlaying lesion masks, but whether this also applies to structural connectomes is unknown.^30^ These studies only compared a subset of metrics from direct and indirect approaches, however. The proliferation of other tract-based, parcel-based and network-based metrics from these two approaches means that more comprehensive comparisons between direct and indirect metrics are needed.

Another major difference across studies is whether connectivity is defined based on known white matter tracts or based on plausible connections between grey matter regions of interest (ROIs). For tract-based analyses, the structure of a tract can be quantified using different measures. The most common properties are FA, axial diffusivity (AD), radial diffusivity (RD) and mean diffusivity (MD) averaged from the voxels of tracts.^18^ Although discrepancies among these conceptually different metrics are expected,^31^ as a practical matter, it is unknown whether they are analogous. Many studies have used these four metrics together and found they produce similar results.^32-34^ However, when comparing these micro-structural metrics with morphometric macro-structural metrics (e.g. volume), the agreement between the measures is not very strong.^21,35,36^ For indirect measures derived from lesion masks, there is disagreement about whether tract disconnection information is more informative than lesion load (i.e. percent damage).^23^ It may be that both measures are equally effective at predicting symptoms,^37^ or that neither independently contributes to deficit prediction,^38^ perhaps because they are strongly correlated with grey matter damage.

An alternative to the tract-based approach is a parcel-based connectome approach in which a matrix is constructed from the structural connections between pairs of grey matter parcels. Analyses can then evaluate the relationship between these connections and behavioural deficits using either mass univariate or multivariate methods. Graph theory also provides dozens of metrics for representing the mathematical properties of the network defined by the connectome matrix.^39^ In addition to different approaches (discussed above), studies construct parcel-based connectome networks using different network scales. For example, if the sample is people with aphasia following left hemisphere stroke and the behavioural symptom measure is language performance, a reasonable analysis strategy is to restrict the analysis to the language network. Indeed, this is a common strategy in CLSM studies of post-stroke aphasia.^40,41^ But since regions outside the canonical language network might be recruited during recovery, it would also be reasonable to use whole-brain connectomes, as other studies have done.^42,43^ Because graph theory metrics describe mathematical properties of the network as a whole, the scale of the brain network under analysis has major conceptual implications. For example, it may be that naming recovery is associated with different aspects of the whole-brain network and the local language network.^42^ To our knowledge, there is no post-stroke aphasia study that compares the same metrics at different network scales.

In sum, a broad range of metrics have been used for CLSM. Although a discrepancy among the biophysically and anatomically different metrics is expected, it is quite possible for two very different metrics to converge as, for example neural activity measured by BOLD signal and by electrophysiology are highly convergent.^44^ Whether CLSM metrics are similar has not been evaluated. To address the gap, we first conducted a systematic review to identify CLSM approaches that are currently in use. The different processing choices included image modality (DWI versus lesion mask), network scale (whole brain versus ROIs) and metric type (e.g. FA versus tract volume; small worldness versus transitivity). Second, we calculated these metrics using data from a previous study of 50 participants with chronic aphasia following left hemisphere stroke. Correlations between these metrics were first examined. Ideally, if two different metrics are capturing the same underlying construct—‘white matter integrity’—then they should be very strongly correlated.

To provide a comprehensive picture of the consequences of using these different metrics, we then conducted a ‘multiverse’^45,46^ of CLSM analyses of naming and comprehension impairment. Multiverse analysis involves systematically reporting the outcomes of all reasonable analyses choices, providing an assessment of the consistency of the results and identifying the critical analysis decision points.^47-50^ In this case, it allows us to illustrate how inferences about the effect of connectome disruption on naming or comprehension would be impacted by different implementations of CLSM. In these analyses, the critical issue is not which effects are significant, but whether the pattern of results is the same or different across CLSM implementations. In other words, to what extent the same inferences could be drawn from different implementations of CLSM.

## Materials and methods

### Systematic literature review

To identify the most common CLSM methods in current use, we performed a systematic literature review of CLSM studies of aphasia published in the last decade (from January 2012 to December 2023). The systematic review procedure is shown in Supplementary Fig. 1. Relevant CLSM articles were selected from PubMed (https://pubmed.ncbi.nlm.nih.gov/). The key words included ‘stroke; language; graph theory/connectome symptom mapping/white matter tracts/structural network/disconnection’. Other relevant articles were added if they could not be identified from the search. To be included, articles must have reported a group-based statistical study of language processing, have used lesion masks or diffusion MRI to investigate white matter connections^51^ and have tested participants who were adult stroke survivors. The number of connections/tracts studied was not an exclusion criterion. Based on their content, the identified articles were further divided into two categories: tract based and parcel based.

The ‘tract-based’ articles were those that included analysis of anatomically pre-defined tracts, such as the arcuate fasciculus (AF) and uncinate fasciculus (see Supplementary Table 1 for details of the 43 tract-based articles). The ‘parcel-based’ articles were those that included analysis using connectome information between grey matter parcels, such as connection strengths or graph theory metrics derived from connectomes (see Supplementary Table 2 for details of the 32 parcel-based articles). Table 1 shows a summary of the key properties of the methods from these articles, which was used to guide our analyses.

**Table 1:** Summary of CLSM literature review

		Tract based			Parcel based	
Number of articles		43			32	
Sample size	Median (SD)	34 (96)			69 (136)	
	Range	7–503			24–818	
Approach	Indirect	11			7	
	Direct	24			25	
	Both	8			0	
Tract/network of Interest	**AF**	25	CC	2	**Whole brain**	20
	**UF**	24	MdLF	2	**Language**	7
	**IFOF**	22	EC	2	Both	5
	**ILF**	20	CST	1		
	**SLF**	11	FST	1		
	**FAT**	11				
Direct metrics	**FA**		26		2	
	**RD**		8			
	**MD**		8			
	**AD**		7			
	**Volume**		6			
	**Number of streamlines**		1		22	
	Fibre density/cross-section		2			
	Axonal water fraction		1			
	Quantitative anisotropy		1			
	HMOA		1			
	Spin distribution function		1			
	Streamline density				1	
Indirect metrics	**Lesion load**		14		1	
	**Number of streamlines (virtual)**				6	
	Image intensity		2			
	Connection status		2			
	Damage probability		2			
	Max damage percentage		1			
Graph theory metrics	**Shortest/characteristic path length/efficiency**				8	
	**Strength/degree**				5	
	**Transitivity/cluster coefficient**				4	
	Betweenness				3	
	**Rich club**				2	
	Controllability				2	
	**Modularity**				1	
	**Small worldness**				1	
	Long-range fibre ratio				1	
	Bypass				1	
	Participation coefficient				1	
	Module size				1	
	Community affiliation index				1	
	Fragmentation index				1	

Bold font indicates the variables used in our analysis. CLSM, connectome lesion-symptom mapping; SD, standard deviation; FA, fractional anisotropy; RD, radial diffusivity; AD, axial diffusivity; MD, mean diffusivity; HMOA, hindrance modulated orientational anisotropy; UF, uncinate fasciculus; AF, arcuate fasciculus; IFOF, inferior fronto-occipital fasciculus; ILF, inferior longitudinal fasciculus; SLF, superior longitudinal fasciculus; FAT, frontal aslant tract; CC, corpus callosum; MdLF, middle longitudinal fasciculus; EC, external capsule; CST, corticospinal tract; FST, frontal striatal tract.

Prior CLSM studies of picture naming have implicated four major white matter tracts: AF, inferior fronto-occipital fasciculus (IFOF), inferior longitudinal fasciculus (ILF) and uncinate fasciculus (UF) using both direct and indirect measures after controlling total lesion size.^23,29,32,37,52-55^ Relevant studies also found evidence that direct (without controlling total lesion size) and indirect (cf. Hope *et al.*^38^) connectomes of whole-brain or language network predicted naming impairments.^40,56,57^ Node strength calculated using direct or indirect whole-brain connectomes was the most reliable graph theory measure when predicting naming deficits in aphasia.^16,56,58^ Other graph theory measures, such as betweenness, transitivity, efficiency (indirect results only) and rich club coefficient, modularity and community number (direct results only without controlling total lesion size), need to be further validated.^56,59,60^ A multiverse analysis was done to evaluate to what extent results are consistent across different implementations of CLSM.

### Participants

The data for these analyses came from baseline measurements of participants who were recruited for aphasia treatment studies and have been described in previous publications.^61-64^ The inclusion criteria were chronic aphasia following a single left hemisphere stroke and English native speakers. Exclusion criteria were history of neurodegenerative disorder, encephalopathy, brain cancer and mental disorders. Fifty participants (33 males, 44 right handed) were recruited at the University of Cincinnati and the University of Alabama at Birmingham. The mean age was 52 years (SD = 15, range = 23–85), and mean time since stroke was 39 months (SD = 38, range = 2–168). All participants completed the language and comprehension assessments (Boston Naming Test, Token Test) and MRI scanning. The mean interval between the administration of the assessments and the MRI scan session was 7 days (SD = 27, range = 0–151). The mean naming score was 34 points (SD = 20, range = 0–60; maximum = 60; Supplementary Fig. 2), and the mean Token Test score was 24 points (SD = 13, range = 4–44; maximum = 45; Supplementary Fig. 2). Age and time since stroke were not significantly correlated with the naming or Token Test scores (|*r*| < 0.06, *P* > 0.65). Mean lesion volume was 91 cc (SD = 65, range = 2–263). Lesion overlap (Supplementary Fig. 3) was typical for aphasia following left middle cerebral artery stroke.^21,52^

### MRI acquisition and pre-processing

T_1_-weighted and DWI sequences were acquired using Philips or Siemens 3T scanners (additional details are available in Adezati *et al.*^61^ and https://osf.io/3snc7/). The parameters for each imaging sequence were as follows: (i) T_1_ from Philips Achieva: *n* = 25; TR = 8100 ms, TE = 3.7 ms, FOV = 25 × 21 × 18 cm, flip angle = 8°, matrix = 252 × 211, slice thickness = 1 mm; (ii) T_1_ from Siemens Allegra: *n* = 18; TR = 2300 ms, TE = 2.17 ms, FOV = 25.6 × 25.6 × 19.2 cm, flip angle = 9°, matrix = 256 × 256, slice thickness = 1 mm; (iii) T_1_ from Siemens Prisma: *n* = 7; TR = 2300 ms, TE = 3.37 ms, FOV = 25.6 × 25.6 × 19.2 cm, flip angle = 9°, matrix = 256 × 256; slice thickness = 1 mm; (iv) DWI from Philips Achieva: *n* = 25; TR = 9403 ms, TE = 69 ms, FOV = 18 × 16.1 cm, voxel size = 1.9 × 1.9 × 2.38 mm, flip angle = 90°, directions = 32, *b* = 800 s/mm^2^; (v) DWI from Siemens Allegra: *n* = 18; TR = 9400 ms, TE = 89 ms, FOV = 24 × 24 cm, voxel size = 2.5 × 2.5 × 2.5 mm, flip angle = 90°, directions = 32, *b* = 800 s/mm^2^; and (vi) DWI from Siemens Prisma: *n* = 7; TR = 5500 ms, TE = 69 ms, FOV = 24.4 × 24.4 cm, voxel size = 2 × 2 × 2.6 mm, flip angle = 90°, directions = 64, b = 1000 s/mm^2^. Scan sequence was included as a dummy-coded covariate to control for any differences arising from the scanning protocols.

As in our previous studies with this data set,^61,64^ lesions were automatically identified from T_1_ images using the LINDA package,^65^ then manually inspected and modified to eliminate errors in segmentation.

### Tract-based connectomes

**Figure 1:**
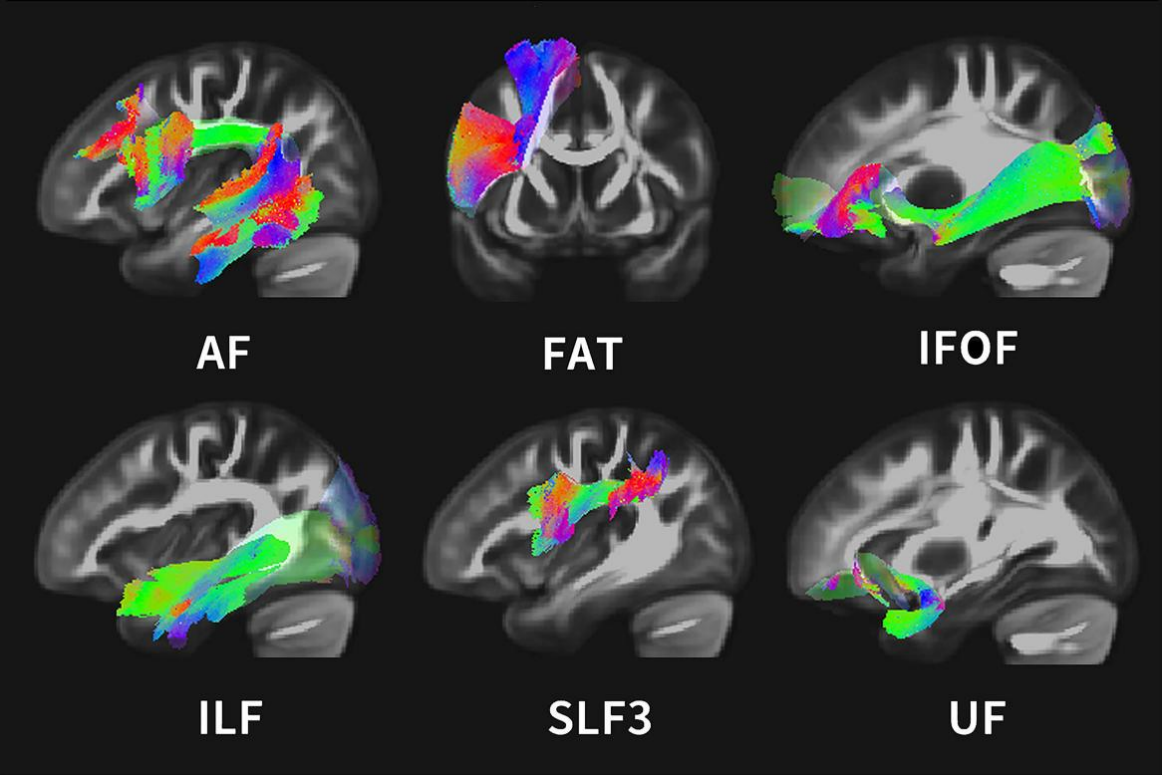
**Visualization of tracts of interest.** Six language-related tracts were chosen from the literature for the analyses. The figure shows shapes of the tracts from the HCP atlas. AF, arcuate fasciculus; FAT, frontal aslant tract; IFOF, inferior fronto-occipital fasciculus; ILF, inferior longitudinal fasciculus; SLF, superior longitudinal fasciculus; UF, uncinate fasciculus.

The tracts and metrics of interest were selected based on the systematic review of tract-based CLSM articles (see Table 1). Six language-related tracts were included: AF, frontal aslant tract (FAT), superior longitudinal fasciculus (SLF) III, ILF, IFOF and UF (see Fig. 1) using the HCP1065 atlas.^66^

‘Indirect’ connectomes were derived from the overlap between a lesion mask and the HCP1065 atlas. The lesion percentage of a tract was calculated as the percentage of the tract that fell within the lesion mask. We also included another metric, disconnection percentage: the percentage of a tract’s streamlines that pass through the lesion mask. This was calculated using the Lesion Quantification Toolkit.^22^

‘Direct’ connectomes were derived from individual DWI images and processed using DSI studio (June 2020). First, they were resampled into 2 × 2 × 2 mm and were eddy current corrected using FSL’s ‘eddy’ tool. Next, images were reconstructed using the ‘Q-space diffeomorphic reconstruction’ method (diffusion sampling length ratio = 1.25).^67^ This method used a model-free approach, ‘generalized Q-sampling imaging’, and transformed the images to MNI space. Tracts were constructed using a deterministic fibre tracking algorithm,^68^ seeded from the corresponding white matter regions in the HCP atlas (5 000 000 iterations with random parameters: anisotropy threshold = 0.5–0.7; angular threshold = 15–90°; step size = 0.5–1.5 voxel distance). The streamlines were compared with corresponding tracts’ streamlines from HCP atlas. Only matched streamlines were retained as specific tracts (tolerance for bundle variation = 16; iterations of topology-informed pruning = 16). The AF was not able to be constructed for seven participants. The IFOF was not able to be constructed for 24 participants. Most of these tractography failures appeared to be due to complete disconnection of the tract (67% for IFOF failures and 86% for AF failures); the others may be due to individual differences in tract anatomy that made the AF or IFOF hard to reconstruct using the current tractography algorithm. As a quality control, we checked all the output tracts and visualized the first two cases (Supplementary Fig. 4), which appeared as expected.

The direct metrics were FA, MD, AD, RD, volume and number of streamlines for each tract. FA, MD, AD and RD were calculated by averaging the values of all the voxels within each tract. Volume was calculated by multiplying the number of voxels within each tract with the voxel size. The distributions of all the direct and indirect metrics are shown in Supplementary Fig. 5. Most metrics did not show strong floor or ceiling effects, except for disconnection percentage, which tended to be bimodal with most values near 1.0 (complete disconnection) or 0.0 (no disconnection).

### Parcel-based connectomes

Consistent with most studies in our systematic review, we adopted probabilistic tractography and generated number of streamlines as the direct connectome metric (Table 1). ‘Direct’ DWI images were processed by the FDT of FSL.^69^ Images were first corrected for head motion and eddy currents. Then diffusion parameters’ Bayesian estimation for each voxel was performed using FSL FDT’s bedpost. Before the tractography, the Automated Anatomical Labeling (AAL3) atlas^70^ was transformed to individual T_1_ native space using the Clinical Toolbox,^71^ which accounts for anatomical deformation caused by lesions. The atlas and lesion masks were further linearly co-registered to individual DWI space using the B0 image as a reference. Finally, probabilistic tractography was performed using FDT’s probtrackX with default tracking parameters (number of individual streamlines drawn in each voxel = 5000; maximum number of steps = 2000; step length = 0.5 mm; curvature threshold = 0.2; fibre volume threshold = 0.01) between all the 166 AAL3’s parcels. Lesion territory was excluded to prevent erroneous streamlines from being reconstructed. For each pair of parcels, the number of streamlines connecting them was generated after correcting for parcels’ distance and volume.

‘Indirect’ parcel-based connectomes were generated by overlaying binarized and spatially normalized lesion masks on the aggregated streamlines from all tracts within the HCP1065 atlas and removing any streamlines that pass through the lesion mask from the aggregated ones using the Lesion Quantification Toolkit^22^ (with the following modified DSI studio parameters: connection criteria for parcels = end, connection criteria for lesion = pass). For each participant, the number of preserved streamlines was calculated between all the pairs of the AAL3 parcels.

Our systematic review found that both whole-brain and language network CLSM have been used; therefore, in addition to the whole-brain connectomes with 166 parcels, direct and indirect language connectomes were constructed using a subset of 14 left hemisphere language-related parcels.^8,40^ These parcels were as follows: the pre- and post-central gyri, supramarginal and angular gyri, insula, middle and inferior frontal gyri (opercular, triangular and orbital segments), superior, middle and inferior temporal gyri and temporal pole (superior and middle segments).

### Graph theory metrics

Graph theory metrics were selected based on the systematic review of published parcel-based CLSM articles (see Table 1). Six common metrics were generated from each of four connectomes (direct and indirect, whole-brain and language network) using the Brain Connectivity Toolbox^39^: characteristic path length, modularity, mean rich club coefficient, strength, small worldness and transitivity (see Supplementary Table 3). The detailed procedure was the same as in our previous study with this data set.^61^ First, all the connection weights were normalized between 0 and 1 to calculate most of the graph theory metrics. Then they were converted to a length matrix, defined as the inverse of connection weights. Finally, the length matrix was converted into a distance matrix, showing the shortest path between regions to generate characteristic path length. The nodal strength was averaged to generate a global value. The rich club coefficients at different degree distributions were averaged. The network was randomized 100 times to calculate small-world propensity.

### Statistical analyses

Similarities among tract-based metrics were assessed across participants using bivariate correlations for each tract. A step-wise regression was used in the multiverse analysis to predict naming or Token Test scores. The null model included total lesion size and a dummy-coded scanning protocol variable, and then each tract’s metric was entered into the model using the ‘add1’ function of the package ‘stats’ in R. We evaluated each metric individually because our focus is on the effects across metrics, not on comparing the tracts to one another. We controlled for total lesion size because it was highly correlated with the naming (*r* = −0.51, *P* = 0.0002) and Token Test scores (*r* = −0.62, *P* = 0.000002). Improvement in model fit (*r*^2^) and corresponding *P*-value were calculated for each metric. We did not perform any familywise error correction because our goal was to evaluate the similarities of different metrics under different processing choices, so controlling false positive brain–behaviour associations was not relevant. For this illustrative purpose, we used a traditional threshold (*P* < 0.05) to display the result pattern.

To investigate the similarity of parcel-based direct and indirect connectomes, bivariate correlations were performed for all parcel pairs for each participant’s whole-brain or language connectome. Because the connectomes are often skewed, we used Kendall non-parametric rank correlation. Sparse canonical correlation analysis for neuroimaging (SCCAN; implemented in the ‘lesymap’ package)^72^ was used in a multiverse analysis to evaluate how the pattern of parcel-based connectome damage is associated with language deficits. We used SCCAN here because this multivariate model is suitable for high-dimensional data (i.e. the connectome data); we used OLS multiple regression for other analyses that only examined a small number of predictors (i.e. tract-based and graph theory measures). This is also more consistent with how CLSM is conducted in the field: SCCAN would be used with high-dimensional connectomes while regression would be used for a handful of connectivity measures.^32,57,60^ The dependent variable was the residual of performance on the Boston Naming or Token Test, regressing out total lesion size and the dummy-coded confounder of scanning protocol. Four-fold cross-validation (CV) was used to evaluate goodness of model fit, with statistical significance assessed by correlation between observed and predicted behavioural scores.

**Figure 2:**
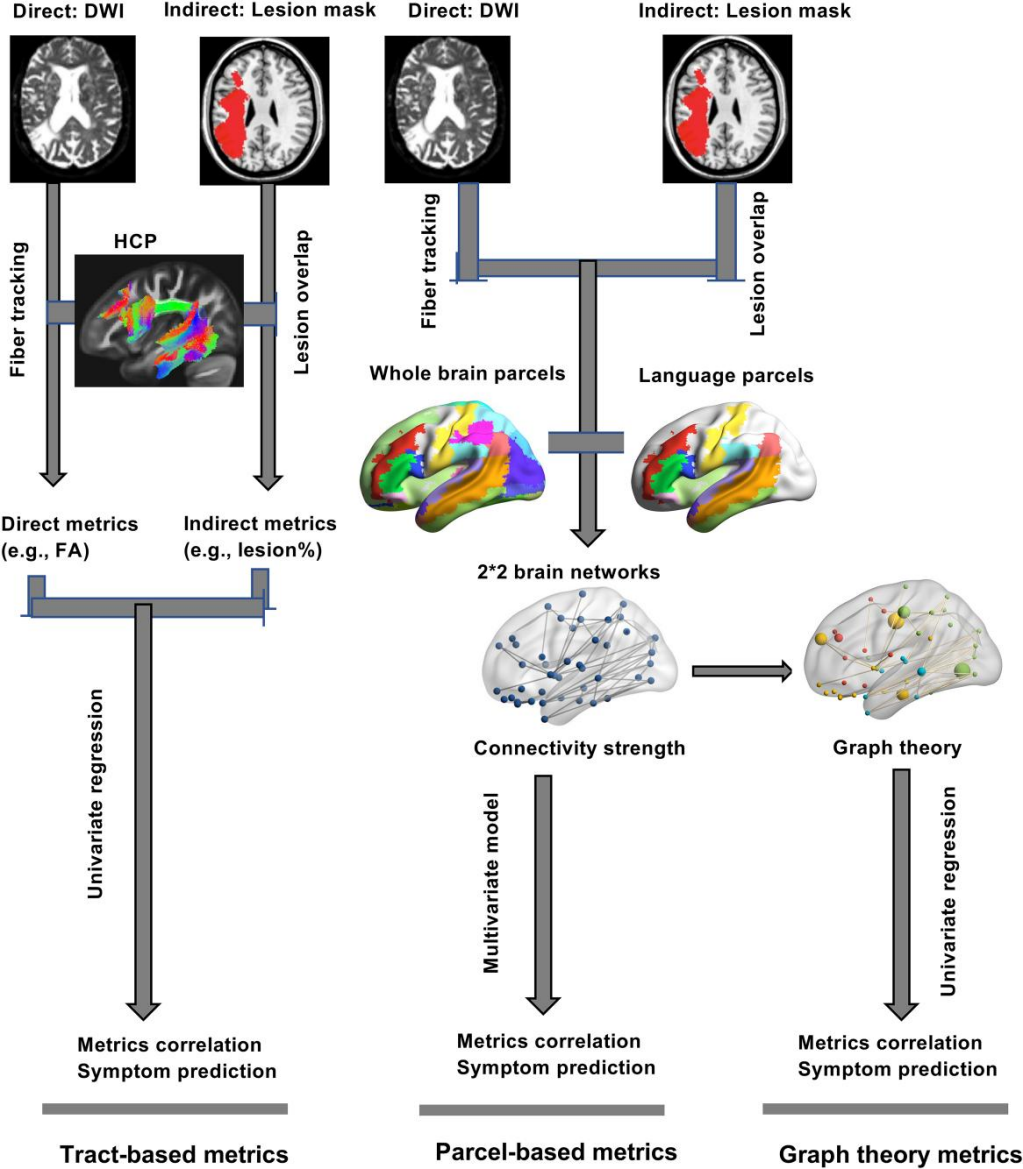
**A flowchart of generating metrics and conducting CLSM analyses.** For tract-based metrics, direct/indirect metrics were generated by overlaying DWI/lesion masks into the HCP white matter atlas and then were used for correlation and prediction analyses. For parcel-based metrics, direct/indirect connectivity was generated in the whole-brain/language parcels and used for correlation and prediction analyses. For graph theory metrics, direct/indirect plus whole-brain/language-related metrics were generated based on the four different kinds of connectivity and were used for correlation and prediction analyses. DWI, diffusion-weighted imaging; HCP, Human Connectome Project; FA, fractional anisotropy.

Similarity of graph theory metrics derived from direct and indirect connectomes was assessed by bivariate correlations across participants, separately for language network and whole-brain graph theory metrics. Just as with the tract-based metrics, the multiverse analysis used step-wise regression controlling for total lesion size and a dummy-coded confounder of scanning protocol to test the association between graph theory metrics and language production and comprehension deficits (see Fig. 2 for the flowchart of the analyses).

### Ethics statement

This study was conducted according to the guidelines of the Declaration of Helsinki and approved by the institutional review boards of the University of Cincinnati and the University of Alabama at Birmingham and the PPLS Research Ethics panel of the University of Edinburgh. All participants gave written informed consent prior to participation.

## Results

### Tract-based measures

**Figure 3:**
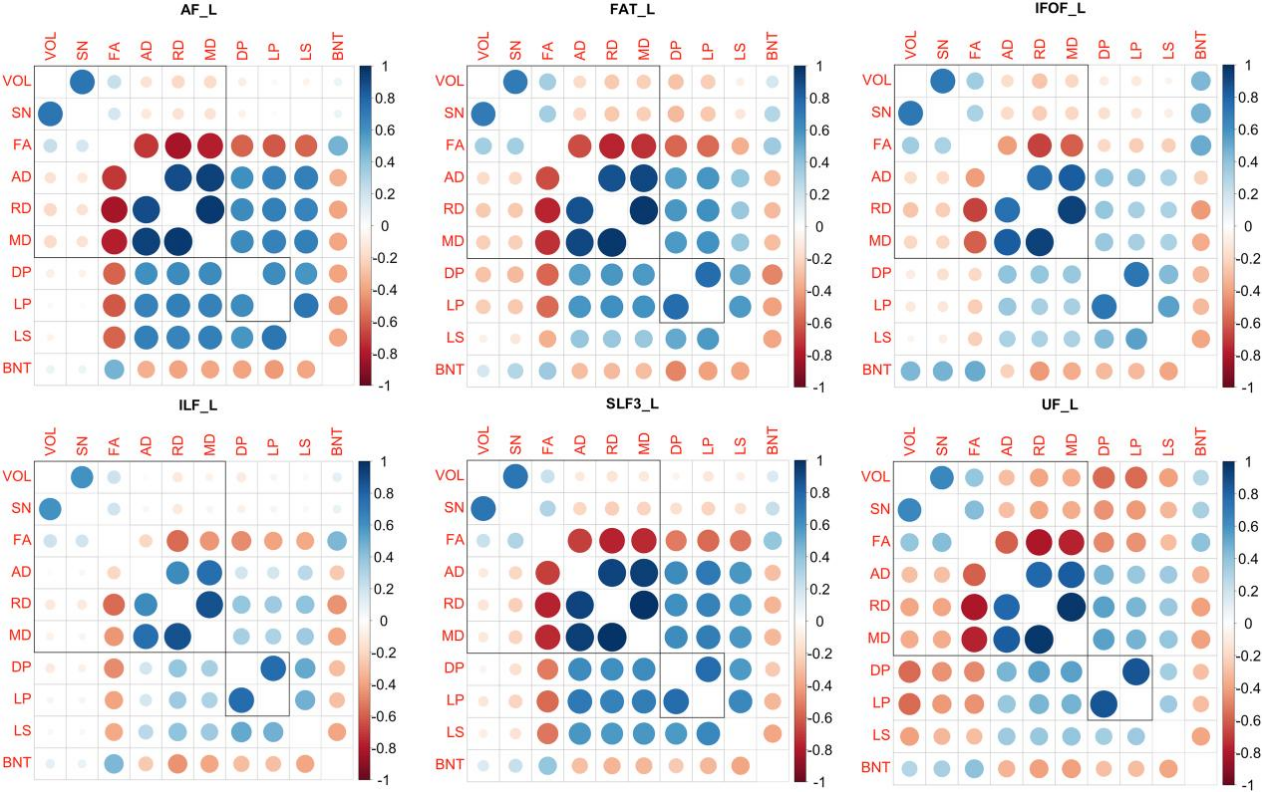
**Correlation values among tract-based measures, lesion size and naming performance.** The two square outlines distinguish direct and indirect metrics. Pearson correlations were based on *N* = 50 participants except for AF (*N* = 43) and IFOF’s (*N* = 26) direct metrics. VOL, tract volume; SN, streamline number; FA, fractional anisotropy; AD, axial diffusivity; RD, radial diffusivity; MD, mean diffusivity; DP, disconnection percentage; LP, lesion percentage; LS, overall lesion size; BNT, Boston Naming Test; AF, arcuate fasciculus; FAT, frontal aslant tract; IFOF, inferior fronto-occipital fasciculus; ILF, inferior longitudinal fasciculus; SLF, superior longitudinal fasciculus; UF, uncinate fasciculus.

First, we examined the similarity among different tract-based metrics (Fig. 3). For the AF, FAT and SLF3, the two macro-structural direct metrics (total volume and number of streamlines) highly correlated with each other (|tau| > 0.69, *P* < 1e^−12^). The other micro-structural direct metrics (FA, AD, RD and MD) correlated with the indirect metrics (tract disconnection and tract lesion percentage), lesion size and naming performance (tau| > 0.26, *P* < 0.02). For the IFOF and ILF, the macro-/micro-structural direct and indirect metrics highly correlated within themselves (|tau| > 0.20, *P* < 0.04). For the UF, all the metrics were moderately or strongly correlated (|tau| > 0.29, *P* < 0.005). However, note that a statistically significant correlation is only sufficient to reject the null hypothesis that the metrics are completely unrelated; correlations in the 0.2–0.3 range indicate only 5–10% shared variance and may produce very different results. This was explored in a multiverse analysis.

**Figure 4:**
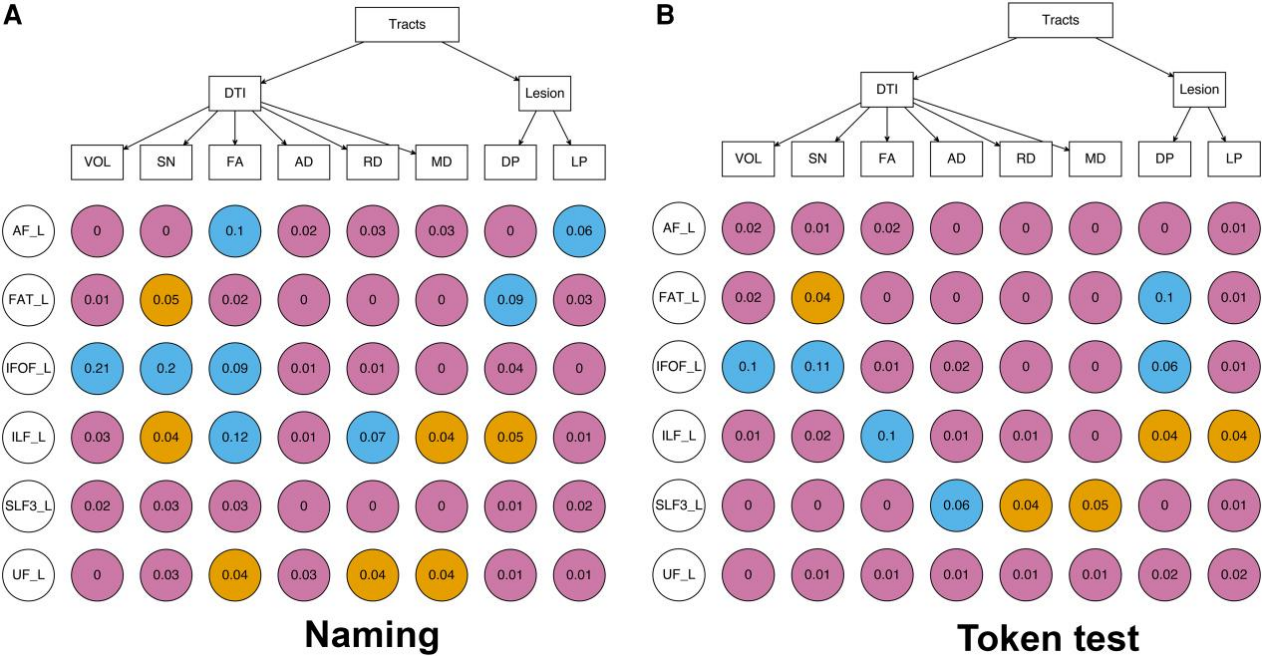
**Multiverse analysis of tract-based measures.** First, the basic regression model was built using the total lesion size and scanning protocol to predict behavioural symptoms within 50 participants except for AF (*N* = 43) and IFOF’s (*N* = 26) DTI metrics. Then, each tract’s metric was added into the basic model. Each data point is the *R*^2^ change when each tract measure was added into a base model of naming (**A**)/token test (**B**). Blue colour indicates *P* < 0.05; yellow colour indicates *P* < 0.1; violet colour indicates *P* > 0.1. VOL, volume; SN, streamline number; FA, fractional anisotropy; MD, mean diffusivity; AD, axial diffusivity; RD, radial diffusivity; DP, disconnection percentage; LP, lesion percentage; AF, arcuate fasciculus; FAT, frontal aslant tract; IFOF, inferior fronto-occipital fasciculus; ILF, inferior longitudinal fasciculus; SLF, superior longitudinal fasciculus; UF, uncinate fasciculus.

Multiverse analysis was performed to evaluate the consistency of measures’ associations with naming deficit after controlling for total lesion size and scanning protocol (Fig. 4A). None of the tracts showed a consistent pattern across different metrics: each tract had at least two marginally or statistically significant effects and at least three non-significant effects (except for SLF3, which had no significant or marginal effects). ‘Indirect’ measures based on lesion masks tended not to produce statistically significant effects, except for AF and FAT (though different indirect measures were significant for these tracts). To validate our findings, we repeated these analyses using the token test, a measure of language comprehension deficit. The token test results showed a similarly inconsistent pattern (Fig. 4B).

### Parcel-based connectomes

**Figure 5:**
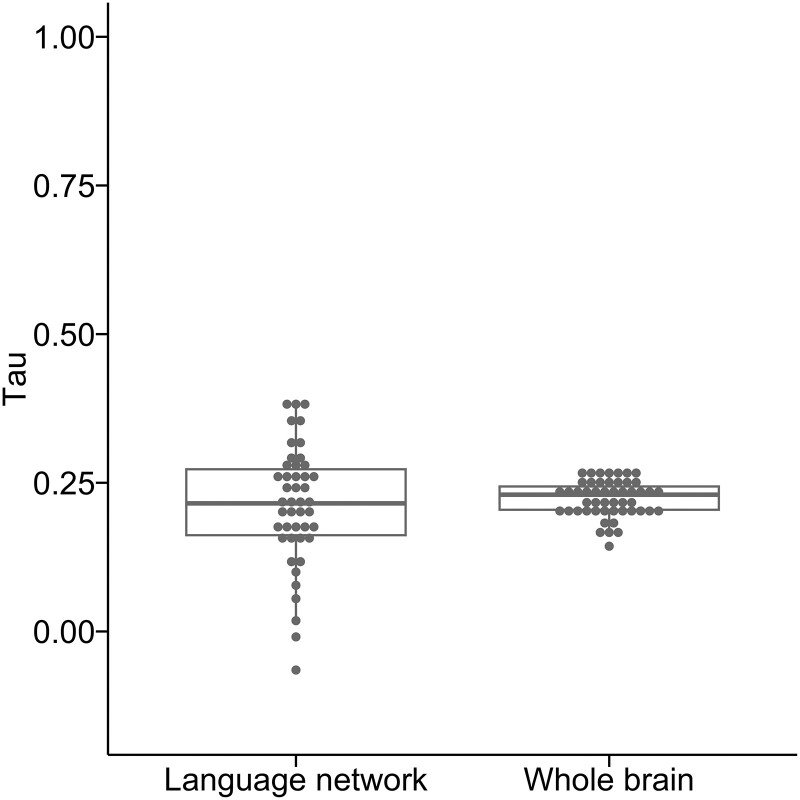
**Kendall correlation values between direct and indirect parcel-based connectomes for each participant** (language network: *n* = 91; whole brain: *n* = 13 695). A paired test was done to compare the two conditions across 50 participants (*t* = −0.67, *P* = 0.50).

For parcel-based connectomes, correlations between direct and indirect connectomes (Fig. 5) were low among the language-related parcels (median = 0.22; range = −0.06–0.39) and more broadly in the whole-brain connectomes (median = 0.23; range = 0.14–0.27), with no statistically significant difference between them (*t* = −0.67, *P* = 0.50).

None of the multivariate CLSM analyses could predict naming scores from parcel-based connectomes (CV *r* < 0.19, *P* > 0.18; Supplementary Table 4). The results were similar for token test: none of the token test models were significant (*r* < 0.20, *P* > 0.16; Supplementary Table 4).

### Graph theory measures

The graph theory measures derived directly from DWI were only weakly correlated with analogous measures derived indirectly from lesion maps: of the 12 total measures (6 graph theory metrics, calculated separately for the whole-brain network and the language network), 10 measures had correlations < 0.3 (see Table 2).

**Table 2:** Correlation values between direct and indirect graph theory measures

	Whole brain	Language network
Characteristic path length	−0.03	−0.02
Modularity	0.04	−0.23
Rich club	0.36^a^	0.32
Strength	0.27^a^	0.53^a^
Small world	0.16	−0.09
Transitivity	−0.19	−0.41

^a^One-tail *P* < 0.05. Because the direct and indirect measures are nominally measuring the same aspect of brain connectivity, the magnitude of the correlation reflects convergent validity. The statistical significance is not informative because the null hypothesis is not meaningful (i.e. that direct and indirect versions of the same graph theory metric are uncorrelated).

**Figure 6:**
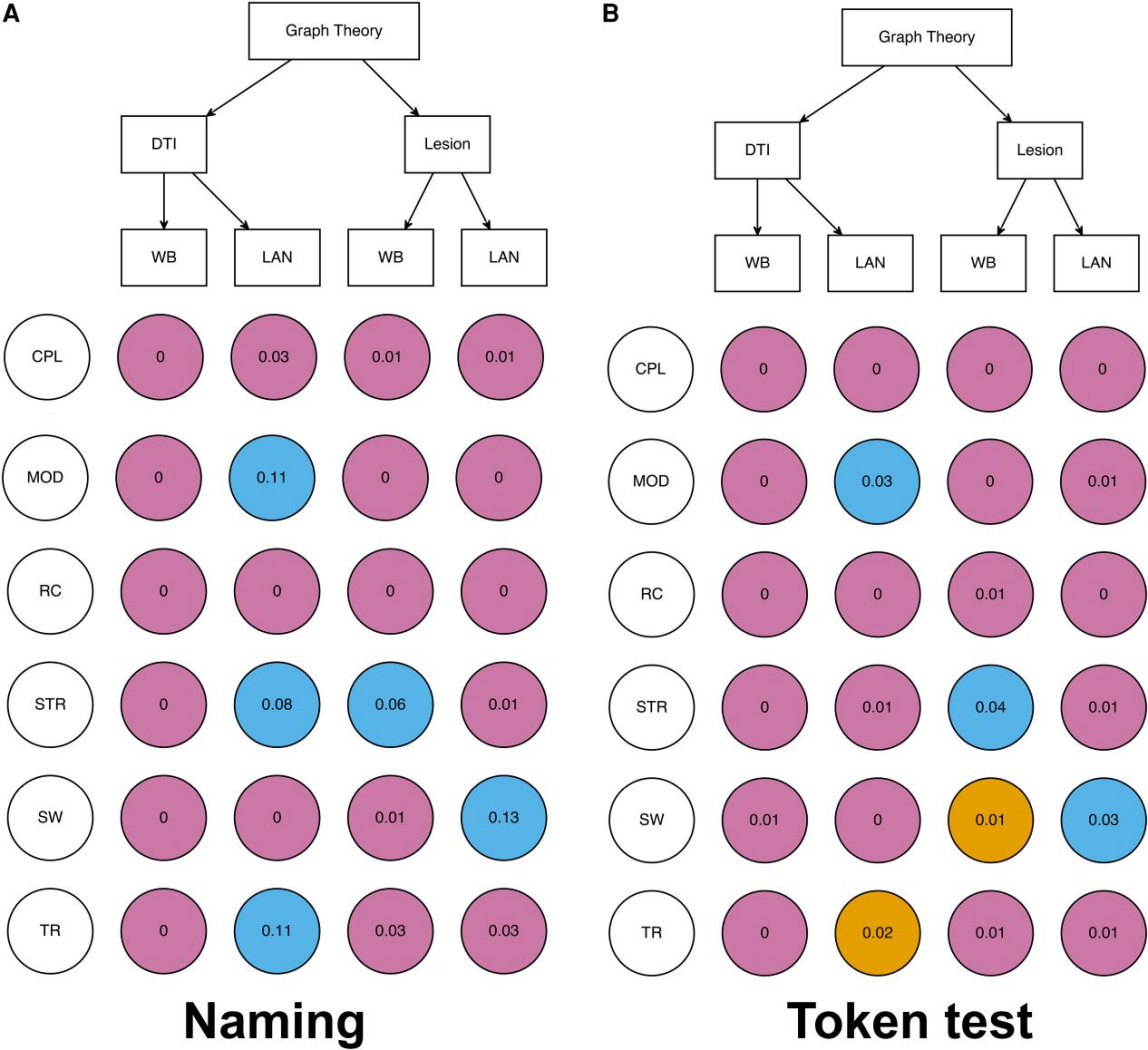
**Multiverse analysis of graph theory metrics.** First, the basic regression model was built using the total lesion size and scanning protocol to predict behavioural symptoms within 50 participants. Then, each graph theory metric in each condition was added into the basic model. Each data point is the *R*^2^ change when each tract measure was added into a base model of naming (**A**) or token test (**B**). Blue colour indicates *P* < 0.05; yellow colour indicates *P* < 0.1; violet colour indicates *P* > 0.1. WB, whole brain; LAN, language network; CPL, characteristic path length; MOD, modularity; RC, rich club; STR, strength; SW, small world; TR, transitivity. The language network was defined as left pre- and post-central gyri, supramarginal and angular gyri, insula, middle and inferior frontal gyri (opercular, triangular and orbital segments), superior, middle and inferior temporal gyri and temporal pole (superior and middle segments).

Multiverse analysis revealed no consistent patterns regarding which graph theory measures were associated with naming performance after controlling for total lesion size and scanning protocol (Fig. 6A). Each measure had at most two marginal or statistically significant effects under four network sets (direct versus indirect, whole-brain versus language network). Only strength showed a significant effect in both of the approaches; however, they were in different network scales. None of the measures showed a consistent statistically significant effect for whole-brain and language networks. Analogous analyses of graph theory measures as predictors of Token Test performance showed a similarly inconsistent pattern of results (Fig. 6B).

## Discussion

In this study, we examined different analysis choices for CLSM: approaches (direct, indirect), network scale (whole-brain, language network) and metric type (tract based, parcel based or network based). Tract-based FA, AD, RD and MD were highly correlated with indirect metrics and total lesion size but not with tract volume and number of streamlines. The connectome strength and graph theory metrics did not have high correlations between direct (DWI) and indirect (lesion mask) networks, neither in the whole brain nor within the language network (the relevant region for this post-stroke aphasia sample). That is, overall, there was low similarity among CLSM metrics.

### Decision points

The present analyses focused on three major choices in CLSM analysis that vary across published CLSM studies: imaging modality, network scale and measure type. We found high discrepancy across each of these factors. In this section, we discuss them in more detail.

#### Approaches

Directly measured and indirectly estimated parcel-based connectomes were only weakly correlated (median tau = 0.22). That is, direct and indirect estimates of white matter connectivity between any two cortical regions (even just those within the language network) were only somewhat similar. From a psychometric perspective, the typical threshold for measures to be considered similar is a correlation above 0.70; that is, two measures of the same underlying construct should be strongly correlated (tau > 0.70).

Intuitively, the direct approach should be more accurate than an indirect approach that is based on overlap between a T_1_-derived lesion map and an atlas. For example, Salvalaggio *et al.*^30^ found that when predicting functional deficits after stroke, functional connectivity derived directly from resting-state fMRI was superior to an indirect estimate based on lesion overlap with a generic functional connectivity map. In our study, neither the correlation nor the regression results showed strong similarity between direct and indirect approaches. This inconsistency could be attributed to inaccuracy of estimations (e.g. presence of crossing fibres for FA) or differences in pre-stroke anatomy, or other factors. Unfortunately, we do not know the ‘ground truth’ here, so it is not clear whether the inconsistency is a problem with one or both of the metrics. It may be tempting to reject indirect connectivity as invalid and only accept DWI-based direct connectivity metrics. However, even the direct metrics lacked consistency: for example, the correlation between tract volume and FA was low. Therefore, both direct and indirect metrics are in need of further validation.

#### Network scale

There is an inherent trade-off when deciding the scope of brain regions for an analysis: the whole-brain network might include irrelevant information, while the smaller language network might miss critical regions, especially since long-term recovery presumably involves some functional reorganization and recruitment of spared brain regions.^33,73^ The correlations between direct and indirect metrics or connectomes were similarly low for the language network and whole brain, suggesting that image modality differences are general across network scales. When using connectomes to predict word production (naming) or sentence comprehension (Token Test) scores, none of the models were significant, irrespective of network scale or image modality. Network scale may be particularly important for graph theory measures because they integrate information across the full connectome. Thus, network scale is a critical factor when interpreting CLSM results.

#### Metric types

Researchers have developed a large number of metrics intended to quantify white matter damage.^22,74^ Here, we examined three different kinds of metrics—tract based, parcel based and network based—each having a different underlying basis or premise. First, white matter tracts have a relatively clear anatomical basis,^7,75^ even though some of the nomenclature, definition and sub-architecture are still controversial (e.g. SLF).^76,77^ Since the anatomical basis is relatively clear, the discrepancies between metrics reflect a measurement difference. In the correlation analysis, the main discrepancy occurred between macro-structural variables (volume and number of streamlines) and micro-structural and indirect variables. We speculate that this is because the measures are differentially sensitive to the structural damage of the white matter, though the mechanism of this is still unknown (e.g. the biological meaning of streamline number may be influenced by features of the pathway and experimental conditions^17^). The regression results were even more different (e.g. which white matter tracts are critical for picture naming depends on which metric is used).

Second, the parcel-based connectomes showed a divergence between direct and indirect connectivity. This is (at least) partly a result of indirect metrics lacking information about connectivity outside of the lesion territory. In addition, these connectomes inherently depend on cortical parcellation: the Brodmann, AAL and Harvard-Oxford atlases will produce somewhat different connectomes for the same individual. Different parcellations produce graphs with different numbers of nodes and edges, which has a substantial impact on graph theory metrics, and can affect analytical outcomes such as accuracy of symptom predictions.^78,79^ Many atlases are defensible choices, though some are likely to be better than others (e.g. connectivity atlases such as the Brainnetome Atlas and HCP-MMP may be better for disconnectome mapping). More research is needed to understand the consequence of that choice and to identify an optimal atlas.

Finally, unlike tract-based measures and parcel-based connectomes, graph theory metrics are grounded in mathematics rather than neuroanatomy.^80^ It makes sense to expect divergence between dozens of metrics because each of them reflects a different specific property of information spread in the network.^39^ However, as noted by Hallquist and Hillary,^80^ articles often report one or a few idiosyncratic graph theory metrics motivated by their hypothesis, which precludes meta-analysis or even qualitative evidence accumulation. Reporting multiple metrics is helpful to further understand them.

### Total lesion size

The appropriateness and consequences of controlling for lesion size is a complex issue.^81^ Many measures of connectivity were strongly correlated with lesion size, as were the behavioural outcome measures, which makes lesion size a potentially major confounding variable with substantive impact on the results.^82^ This situation might result from the topography of lesion distribution in post-stroke aphasia.^81,83^ In this study, total lesion size limits possible inferences: if a disconnection measure is highly correlated with total lesion size, then it may not be measurably different from total lesion size and it is impossible to infer that a symptom is a result of connectivity disruption rather than of overall lesion size.

### Sample size

A limitation of this study is the moderate sample size (*n* = 50), which is typical for the field (our systematic review found median sample size was *n* = 45). In the parcel-based connectome analyses, none of the four connectome networks significantly predicted naming impairment. There is no reason to believe that a larger sample could change this—the observed effect size was very small, exceptionally difficult to distinguish from no effect at all.

Conversely, the tract-based and graph theory analyses revealed an inconsistent mixture of statistically significant and non-significant effects. This is related to the broader problem that correlations between direct and indirect metrics were rarely larger than 0.5, indicating that the predictors themselves are inconsistent. Determining an adequate sample size is not possible when the measurement validity has not been established. On a more optimistic note, some of the metrics showed higher consistency between direct and indirect versions (e.g. the consistency of strength was higher than of other graph theory metrics). Therefore, it may be that some metrics are more robust than others, though further validation work is required to establish this.

Sample sizes in LSM studies are strongly limited by practical factors of recruiting and testing participants with brain injuries. There are multiple potential inclusion/exclusion criteria, such as aetiology (stroke, neurodegenerative disorders, surgical resection etc.), damage location (e.g. left hemisphere only or whole brain) and chronicity stage (acute, subacute and chronic). Here we used a moderately strict criterion typical for the field (left hemisphere stroke, chronic stage), which reduces the sample size but provides some control over potentially confounding factors. Optimally managing this trade-off depends on the research question, but may also pose further problems for establishing the comparison of connectivity measures because they may differ across aetiologies or chronicity.

### Future directions

Given the broad choices of CLSM, their low consistency and unknown validity, here are several methodological suggestions for the validation of CLSM results: (i) having distinct discovery and validation samples to assess the robustness of the results; (ii) using other experimental approaches, such as meta-analyses or task fMRI; and (iii) using ROI-based or theory-driven analyses instead of data-driven analyses.

It is also important to note that there are many other decision points in CLSM that were not evaluated or discussed here. Decision points, such as atlas selection, diffusion model and tractography algorithm, are more well-known and broadly discussed in the neuroimaging community; therefore, this study focused on less acknowledged decision points that particularly impact CLSM research. Moreover, many new approaches for connectome analysis are continuously described. For example, individualized connectomes could be constructed from structural MRI based on brain morphology^84^, even though this approach is still not adopted in CLSM. These new approaches should be validated as well before they are widely used. That is, all methodological processing choices need to be systematically explored, possibly using the multiverse analysis approach taken here, in order for knowledge to accumulate.

## Conclusion

In sum, we compared different processing choices that arise when implementing CLSM analyses and found that different approaches (direct versus indirect), network scale (whole-brain versus language network) and metric types (multiple tract/graph theory metrics) all led to highly divergent results. Substantial validation work is necessary for these metrics and, in the meantime, multiverse analysis can help enhance the generalizability of results.

## Supplementary Material

fcae313_Supplementary_Data

## Data Availability

The data and code of this study are available on the OSF project page (https://osf.io/t2r9s/).
