# Supplementary material for: Metric comparison of connectome-based lesion-symptom mapping in post-stroke aphasia
Source: Brain Commun. 2024 Sep 12;6(5):fcae313. doi: 10.1093/braincomms/fcae313 (PMC11420983; doi:10.1093/braincomms/fcae313)
Supplement: fcae313_Supplementary_Data [file fcae313_supplementary_data.pdf]

Supplementary Materials

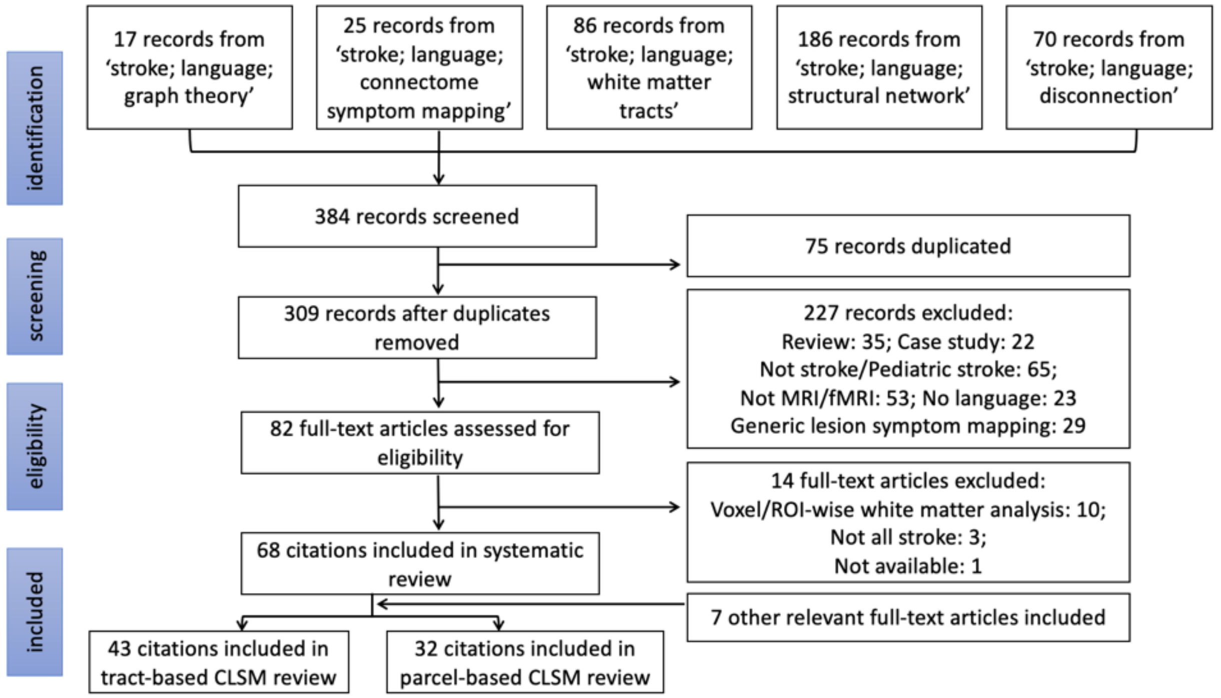

Supplementary Figure 1. The flowchart of connectome-lesion symptom mapping (CLSM) systematic review. A literature review was done before the analyses. 384 Relevant articles were chosen from PubMed using five pairs of keywords. Then 316 articles were excluded and 7 articles were included based on the criteria. Finally, the articles were divided into the tract-based (N=43) and parcel-based groups (N=32).

Supplementary Table 1. The main information of tract-based CLSM articles.

Authors	Participants	Behavior	Imaging modality	Tract index	Tracts of interest
Fridriksson et al., 2013[1]	64 LH chronic stroke aphasia	Diadochokinetic rate, WAB's fluency, auditory word-picture matching	T1	Image intensity	Left 3 segments of AF, IFOF, ILF, UF from Catani et al., 2012[2]
Harvey et al., 2013[3]	10 LH chronic aphasia	Auditory word-picture verification, PPT test	DWI	FA	Left IFOF, ILF, UF from deterministic tracking
Basilakos et al., 2014[4]	76 LH chronic aphasia	WAB fluency, PPT	T1	T1 intensity	Left UF, FAT, ILF, anterior AF from Catani et al., 2012[2]
van Hees et al., 2014[5]	8 LH chronic aphasia	Naming improvement	DTI	Fiber number, generalized FA	Bilateral AF, UF from deterministic tracking
Geva et al., 2015[6]	15 LH middle cerebral artery chronic aphasia	Auditory sentence comprehension, word repetition, object naming, rhyme judgement, homophone judgement	DTI, T2	FA, tract volume, lesion-tract overlap	Bilateral AF from deterministic tracking
Harvey & Schnur, 2015[7]	15 chronic LH middle cerebral artery stroke	Bocked-cyclic naming and word-picture matching	DWI	FA	Left IFOF, ILF, UF from deterministic tracking
Hope et al., 2016[8]	142 chronic LH focal stroke	Fluency and naming T scores of the comprehensive aphasia test	T1	Lesion load, tract disconnection information	Left AF and UF from Thiebaut de Schotten et al., 2021[9] atlas
Ivanova et al., 2016[10]	37 LH chronic aphasia	Comprehension and production at the word and sentence level	DTI	FA, MD, RD, AD	Bilateral AF, SLF, ILF, IFOF, CST from JHU atlas, CC: genu, body, splenium from Juelich Histological atlas

Griffis et al., 2017[11]	43 chronic LH aphasia	Naming, verbal fluency, auditory semantic decisions	T1	Lesion load	Anterior and posterior Bottlenecks of tracts from ICBM-DTI atlas
Li et al., 2017[12]	51 post 1month stroke	Semantic and phonological fluency	DWI, T1	FA, lesion percentage	JHU white matter tractography atlas and bilateral FAT from NeuroVault
Xing et al., 2017[13]	40 LH chronic aphasia	Composite word-level comprehension, composite sentence-level comprehension, PPT	DTI	FA, MD, AD, RD	Left IFOF, ILF, UF, AF from probabilistic tracking on HC
Yang et al., 2017[14]	18 LH stroke aphasia	AQ of aphasia battery of Chinese	DTI	FA	Left IFOF, ILF, SLF, UF from JHU atlas
Forkel & Catani, 2018[15]	18 LH MCA hyperacute aphasia	WAB-AQ	DTI, T1	FA, volume, damage probability	Bilateral AF 3 segments, IFOF, UF, FAT from deterministic tracking; Left AF 3 segments, IFOF, UF, FAT from Tractotron
McKinnon et al., 2018[16]	32 LH chronic aphasia	Semantic and phonological paraphasias of PNT	Diffusion kurtosis imaging	FA, axonal water fraction	Left SLF and ILF from deterministic tracking
L. Zhao et al., 2018[17]	410 acute stroke/transient ischaemic attack	Montreal cognitive assessment: language	DWI, CT for lesion tracing	Lesion load	ICBM-DTI-81 white matter tract atlas
J. Zhang et al., 2018[18]	14 LH subacute stroke	AQ, comprehension, repetition, naming, reading, writing of Aphasia Battery of Chinese	DTI	FA, MD, AD, RD	Left SLF, AF, IFOF, UF, ILF from deterministic tracking
Bonilha et al., 2019[19]	48 LH chronic stroke	A ratio of number of different words between speech entrainment and picture description	T1, T2	Lesion percentage	Left tracts from JHU atlas

Geller et al., 2019[20]	128 LH aphasia>1 month	WAB-AQ, PNT, factors of speech production, semantic cognition	T1/CT	Lesion load, tract disconnection information	Left UF, AF, IFOF from Rojkova et al., 2016[21] atlas
Meier et al., 2019[22]	34 chronic LH ischemic stroke anomia	WAB AQ, BNT, treatment gain	T1, DTI	Spared tissue percentage, FA	Bilateral AF, IFOF, ILF, UF from JHU atlas
Moulton et al., 2019[23]	28 LH ischemic aphasia with thrombolysis treatment	Aphasia rapid test, aphasia handicap score	DTI	Ratio of the affected/unaffected AD	Bilateral AF, IFOF, ILF, UF from deterministic tracking
Blom-Smink et al., 2020[24]	10 LH stroke aphasia	Change in BNT	DTI	FA	Bilateral SLF, IFOF, ILF, MdLF, UF from deterministic tracking
Hartwigsen et al., 2020[25]	12 single embolic chronic stroke	Phonological/semantic stimulation-induced response delay of word decision	DTI	FA	Right SLF, IFOF from Juelich Histological atlas
Hula et la., 2020[26]	42 LH chronic stroke aphasia	Parameters of s and p from semantic-phonological naming error model	Diffusion spectrum imaging	Normalized spin distribution function value	Whole brain tracts from deterministic tracking
Keser et al., 2020[27]	28 LH ischemic aphasia	Number of correct content units and cohesion score from picture descriptions	DWI	FA, RD	Bilateral AF, SLF, FAT, IFOF, ILF, UF from Human Connectome Project white matter atlas
Zyryanov et al., 2020[28]	20 LH stroke	Sentence completion, picture-word interference	DWI	Volume, FA, MD, RD	Left FAT, long segment of AF from deterministic tracking
Gajardo-Vidal et al., 2021[29]	134 LH stroke	Overall score of ACT picture description	T1	Lesion percentage	Left anterior AF, UF from Natbrainlab atlas, FAT from Rojkova et al., 2016[21]
Kim et al., 2021[30]	50 LH subcortical stroke	WAB AQ	T1, FLAIR	Lesion load	11 left tracts from JHU atlas

Kourtidou et al., 2021[31]	25 LH middle cerebral artery chronic aphasia	Boston diagnostic aphasia examination fluency, BNT, Peabody picture vocabulary test, word fluency, comprehension of instructions	DTI	FA, AD, RD	Bilateral AF, SLF ii, SLF iii, temporo-frontal extreme capsule fasciculus, right UF from deterministic tracking
Malherbe et al., 2021[32]	503 stroke	National institutes of health stroke scale factor score	DWI, FLAIR for lesion tracing	Lesion percentage	48 white matter tracts from JHU-ICBM atlas
Martinez Oeckel et al., 2021[33]	123 LH acute ischaemic stroke	Token test	T1, DTI	Maximum percentage of lesion-tract overlap across slices	Left AF and extreme capsule from deterministic tracking
Sihvonen et al., 2021[34]	38 unilateral acute stroke	Change of summary language score	DWI	FA	FAT, IFOF, long segment of AF from deterministic tracking
Soliman et al., 2021[35]	7 subacute non-fluent aphasia	Language, comprehension, naming, repetition, fluency changes of hemispheric stroke scale	Diffusion image	FA, MD	Bilateral AF, FAT, IFOF, ILF, UF from deterministic tracking
B. Zhang et al., 2021[36]	36 subcortical stroke aphasia	AQ, spontaneous speech, comprehension, repetition, naming	T1, DTI	Lesion load, FA	White matter tracts from AALCAT atlas, bilateral UF from deterministic tracking
J. Zhang et al., 2021[37]	29 LH subacute ischemic stroke	Comprehension, naming, repetition, reading, writing, spontaneous speech, aphasia severity of	DWI	Fiber density, FA	Left SLF 3, AF, IFOF, UF, ILF, MdLF, FAT from probabilistic tracking

		Aphasia Battery of Chinese			
Bae et al., 2022[38]	35 aphasia stroke	AQ	DTI	FA, MD, RD, AD	Bilateral AF from deterministic tracking
Braun et al., 2022[39]	34 chronic stroke aphasia	AQ, BNT, potential maximal gain	DTI	FA, MD	CC major/minor, bilateral AF, SLF, ILF, IFOF, UF from deterministic tracking
Sihvonen et al., 2022[40]	39 unliateral acute stroke	Linguistic prosody task	Diffusion image	Volume, FA	CC, tapetum Bilateral 3 AF segments, IFOF, right ILF, UF from deterministic tracking
Zhong et al., 2022[41]	32 LH aphasia stroke	WAB and subset scores, BNT, motor speech evaluation	DWI	Volume, HMOA, lesion load	Bilateral FAT from Natbrainlab Atlas and deterministic tracking
Egorova-Brumley et al., 2023[42]	71 stroke	Average score of BNT and word fluency	DWI	Fiber density, fiber corss-section	72 tracts from deterministic tracking
Olivé et al., 2023[43]	19 chronic aphasia stroke	Phonological and lexical-semantic short term memory	DWI	Volume, FA	Bilateral 3 AF segments, IFOF, ILF, UF
Sihvonen et al., 2023[44]	22 LH early subacute/chronic stroke aphasia	Overall comphrension and production scores	Diffusion image	Quantitative anisotropy	Right tracts of an atlas from deterministic tracking
Soliman et al., 2023[45]	17 LH subacute stroke	Hemispheric stroke scale subsets: compression, naming, repetition, fluency	Diffusion image	FA, MD, RD, AD	Bilateral AF, IFOF, ILF, UF, FAT from deterministic tracking
Xiong et al., 2023[46]	121 chronic ischemic stroke	Phonemic and semantic fluency	T1	Lesion load, disconnection probability	Bilateral 3 AF segments, IFOF, ILF, 3 SLF segments, UF, FAT, FST from BCBToolKit

LH: left hemisphere; WAB: Western aphasia battery; AQ: aphasia quotient; PPT: Pyramids and Palm Trees Test; BNT: Boston naming test; PNT: Philadelphia naming test; DWI: diffusion weighted imaging; DTI: diffusion tensor imaging; FA: fractional anisotropy; MD: mean diffusivity; AD: axial diffusivity; RD: radial diffusivity; AF: arcuate fasciculus; CC: corpus callosum; CST: corticospinal tract; FAT: frontal aslant tract; FST: frontal striatal tract; IFOF: inferior fronto-occipital fasciculus; ILF: inferior longitudinal fasciculus; MdLF: middle longitudinal tract; SLF: superior longitudinal fasciculus; UF: uncinate fasciculus;

Supplementary Table 2. The main information of parcel-based CLSM articles.

Authors	Participants	Behavior	Imaging modality	Connectome index	Node definition	Graph theory index
Kümmerer et al., 2013 [47]	100 LH acute embolic stroke aphasia	Repetition and comprehension scores from Aachen Aphasia Test	T1	Lesion percentage	Language network	
Bonilha et al., 2014[48]	39 LH chronic stroke	WAB AQ and subsets, PNT	DTI	Number of streamlines	Whole brain	
Kuceyeski et al., 2015[49]	41 ischemic acute stroke	Mississippi Aphasia Screen Test, Montreal cognitive assessment	T1 DWI for lesion tracing	Virtual number of streamlines	Whole brain	Node strength
Bonilha et al., 2016[50]	24 LH chronic stroke	Baseline WAB AQ, PNT improvement	DTI	Corrected number of streamlines	Whole brain; language network	Normalized small worldness, averaged nodal betweenness centrality
Gleichgerrcht, Fridriksson et al., 2016[51]	38 LH chronic stroke	Nouns/verbs per minute of 3 picture descriptions	DTI	Corrected number of streamlines	Whole brain	
Gleichgerrcht, Kocher et al., 2016[52]	44 LH chronic stroke	WAB AQ and fluency, comprehension, naming, repetition	DTI	Corrected number of streamlines	Whole brain	Number of rich club nodes
Yourganov et al., 2016[53]	90 LH chronic stroke	WAB AQ and fluency, comprehension, naming, repetition	DTI	Corrected number of streamlines	Whole brain	
Bonilha et al., 2017[54]	67 chronic ischaemic stroke	WAB word comprehension, PPT test	Diffusion MRI	Corrected number of streamlines	Whole brain	

Del Gaizo et al., 2017[55]	92 LH chronic stroke	WAB AQ, WAB fluency	Diffusion MRI	Corrected number of streamlines	Language network	Edge shortest path length
Marebwa et al., 2017[56]	90 LH chronic stroke	WAB AQ and fluency, comprehension, naming, repetition	DTI	Corrected number of streamlines	Whole brain, language network	Modularity, mean node strength, community affiliation index, fragmentation index, number of rich club hubs, module size, nodal intra module degree, nodal participation coefficient
Pustina et al., 2017[57]	53 LH chronic stroke	PNT, WAB AQ, WAB rep, WAB comp	T1	Virtual number of streamlines	Whole brain	Degree, betweenness, local transitivity, local efficiency
Fridriksson et al., 2018[58]	159 chronic stroke	WAB-AQ and subscores, PNT, auditory sentence comprehension, picture description, speech perception, apraxia of speech, PPT	DTI	Corrected number of streamlines	Language network	
Hope et al., 2018[59]	818 stroke	28 comprehensive aphasia test scores	T1	Virtual percentage of streamlines	Whole brain	
Xing et al., 2018[60]	45 chronic LH aphasia	PNT, pseudoword repetition, PPT test	DWI	FA values from healthy controls	Language network	
den Ouden et al., 2019[61]	71 LH ischemic stroke	Argument structure production test, sentence production priming test, sentence comprehension test	DTI	Corrected number of streamlines	Language network	

Griffis et al., 2019[62]	108 sub-acute stroke	Language PCA	T1/T2/T2-FLAIR	Virtual number of streamlines	Whole brain	
Baboyan et al., 2021[63]	71 LH chronic stroke aphasia	WAB repetition, Philadelphia repetition test	Diffusion mono-polar EPI	Corrected number of streamlines	Whole brain	
Dickens et al., 2021[64]	30 LH stroke	Oral reading and phonological assessments	DTI	Binary number of streamlines	Whole brain	
Gleichgerrcht et al., 2021[65]	65 single LH ischemic chronic stroke	PCA of sentence production	Diffusion EPI	Number of streamlines	Whole brain	
Griffis et al., 2021[66]	124 sub-acute stroke	Language PCA involving comprehension, naming and reading	T1/T2/T2-FLAIR	Virtual percentage of streamlines	Whole brain	Edge shortest path length increase
Keator et al., 2021[67]	97 chronic LH stroke	Speech comprehension and production scores from WAB	DTI	Number of streamlines	Language network	
Wilmskoetter et al., 2021[68]	69 chronic LH ischemic stroke aphasia	PNT change	DTI	Normalized number of streamlines	Language network	Edge shortest path length
Adezati et al., 2022[69]	50 LH stroke aphasia	Token test	DWI	Corrected number of streamlines	Whole brain, language network	Cluster coefficient; transitivity; characteristic path length; global efficiency
Blackett et al., 2022[70]	79 chronic stroke aphasia	PNT	Diffusion EPI	Corrected number of streamlines	Whole brain	
Erickson et al., 2022[71]	39 chronic LH stroke	PCA involving lexical production,	DWI	Fractional anisotropy	Whole brain	Bypass elongation/overlap

		auditory comprehension, phonology, semantic				
Matchin et al., 2022[72]	Average of 144 chronic LH ischaemic stroke	Word/sentence comprehension, repetition, expressive agrammatism	DTI	Corrected number of streamlines	Whole brain	
McCall et al., 2022[73]	51 LH chronic stroke	Naming error detection rate	DWI	Apparent fiber density	Whole brain	
Wilmskoetter et al., 2022[74]	68 LH chronic stroke aphasia	Proportion of maximum gain of naming	DTI	Corrected number of streamlines	Whole brain, language network	Global controllability, regional average and modal controllability, node strength, node betweenness centrality
Roth et al., 2023[75]	78 chronic LH stroke	PNT treatment gain	Diffusion EPI	Corrected number of streamlines	Whole brain	Long-range fiber ratio
Zevgolatakou et al., 2022[76]	58 LH stroke aphasia	PCA of speech production	T1/CT	Virtual number of streamlines	Whole brain, language network	Cluster coefficient; characteristic path length; global efficiency
Y. Zhao et al., 2023[77]	68 stroke aphasia	PCA of phonology, semantic, executive function, fluency	DWI	Streamline ratio	Whole brain	
Wilmskoetter et al., 2023[78]	93 chronic LH stroke aphasia	WAB AQ	DTI	Corrected number of streamlines	Whole brain	Global controllability, regional average controllability

LH: left hemisphere; WAB: Western aphasia battery; AQ: aphasia quotient; PNT: Philadelphia naming test; PPT: Pyramids and palm trees test; PCA: principal component analysis; DWI: diffusion weighted imaging; DTI: diffusion tensor imaging; EPI: echo-planar imaging.

Supplementary Table References

1. Fridriksson J, Guo D, Fillmore P, Holland A, Rorden C. Damage to the anterior arcuate fasciculus predicts non-fluent speech production in aphasia. *Brain*. 2013;136(11):3451-3460. doi:10.1093/brain/awt267

2. Catani M, Dell'Acqua F, Vergani F, et al. Short frontal lobe connections of the human brain. *Cortex*. 2012;48(2):273-291. doi:10.1016/j.cortex.2011.12.001

3. Harvey DY, Wei T, Ellmore TM, Hamilton AC, Schnur TT. Neuropsychological evidence for the functional role of the uncinate fasciculus in semantic control. *Neuropsychologia*. 2013;51(5):789-801. doi:10.1016/j.neuropsychologia.2013.01.028

4. Basilakos A, Fillmore PT, Rorden C, Guo D, Bonilha L, Fridriksson J. Regional White Matter Damage Predicts Speech Fluency in Chronic Post-Stroke Aphasia. *Frontiers in Human Neuroscience*. 2014;8. Accessed January 19, 2022. https://www.frontiersin.org/article/10.3389/fnhum.2014.00845

5. van Hees S, McMahon K, Angwin A, de Zubicaray G, Read S, Copland DA. Changes in White Matter Connectivity Following Therapy for Anomia Post stroke. *Neurorehabil Neural Repair*. 2014;28(4):325-334. doi:10.1177/1545968313508654

6. Geva S, Correia MM, Warburton EA. Contributions of bilateral white matter to chronic aphasia symptoms as assessed by diffusion tensor MRI. *Brain and Language*. 2015;150:117-128. doi:10.1016/j.bandl.2015.09.001

7. Harvey DY, Schnur TT. Distinct loci of lexical and semantic access deficits in aphasia: Evidence from voxel-based lesion-symptom mapping and diffusion tensor imaging. *Cortex*. 2015;67:37-58. doi:10.1016/j.cortex.2015.03.004

8. Hope TMH, Seghier ML, Prejawa S, Leff AP, Price CJ. Distinguishing the effect of lesion load from tract disconnection in the arcuate and uncinate fasciculi. *NeuroImage*. 2016;125:1169-1173. doi:10.1016/j.neuroimage.2015.09.025

9. Thiebaut de Schotten M, ffytche DH, Bizzi A, et al. Atlasing location, asymmetry and inter-subject variability of white matter tracts in the human brain with MR diffusion tractography. *NeuroImage*. 2011;54(1):49-59. doi:10.1016/j.neuroimage.2010.07.055

10. Ivanova MV, Isaev DYu, Dragoy OV, et al. Diffusion-tensor imaging of major white matter tracts and their role in language processing in aphasia. *Cortex*. 2016;85:165-181. doi:10.1016/j.cortex.2016.04.019

11. Griffis JC, Nenert R, Allendorfer JB, Szaflarski JP. Damage to white matter bottlenecks contributes to language impairments after left hemispheric stroke. *NeuroImage: Clinical*. 2017;14:552-565. doi:10.1016/j.nicl.2017.02.019

12. Li M, Zhang Y, Song L, et al. Structural connectivity subserving verbal fluency revealed by lesion-behavior mapping in stroke patients. *Neuropsychologia*. 2017;101:85-96. doi:10.1016/j.neuropsychologia.2017.05.008

13. Xing S, Lacey EH, Skipper-Kallal LM, Zeng J, Turkeltaub PE. White Matter Correlates of Auditory Comprehension Outcomes in Chronic Post-Stroke Aphasia. *Frontiers in Neurology*. 2017;8. Accessed March 15, 2022. https://www.frontiersin.org/article/10.3389/fneur.2017.00054

14. Yang M, Li Y, Li J, Yao D, Liao W, Chen H. Beyond the Arcuate Fasciculus: Damage to Ventral and Dorsal Language Pathways in Aphasia. *Brain Topogr*. 2017;30(2):249-256. doi:10.1007/s10548-016-0503-5

15. Forkel SJ, Catani M. Lesion mapping in acute stroke aphasia and its implications for recovery. *Neuropsychologia*. 2018;115:88-100. doi:10.1016/j.neuropsychologia.2018.03.036

16. McKinnon ET, Fridriksson J, Basilakos A, et al. Types of naming errors in chronic post-stroke aphasia are dissociated by dual stream axonal loss. *Sci Rep*. 2018;8(1):14352. doi:10.1038/s41598-018-32457-4

17. Zhao L, Biesbroek JM, Shi L, et al. Strategic infarct location for post-stroke cognitive impairment: A multivariate lesion-symptom mapping study. *J Cereb Blood Flow Metab*. 2018;38(8):1299-1311. doi:10.1177/0271678X17728162

18. Zhang J, Wei X, Xie S, et al. Multifunctional Roles of the Ventral Stream in Language Models: Advanced Segmental Quantification in Post-Stroke Aphasic Patients. *Frontiers in Neurology*. 2018;9. Accessed March 16, 2022. https://www.frontiersin.org/article/10.3389/fneur.2018.00089

19. Bonilha L, Hillis AE, Wilmskoetter J, et al. Neural structures supporting spontaneous and assisted (entrained) speech fluency. *Brain*. 2019;142(12):3951-3962. doi:10.1093/brain/awz309

20. Geller J, Thye M, Mirman D. Estimating effects of graded white matter damage and binary tract disconnection on post-stroke language impairment. *NeuroImage*. 2019;189:248-257. doi:10.1016/j.neuroimage.2019.01.020

21. Rojkova K, Volle E, Urbanski M, Humbert F, Dell'Acqua F, Thiebaut de Schotten M. Atlasing the frontal lobe connections and their variability due to age and education: a spherical deconvolution tractography study. *Brain Struct Funct*. 2016;221(3):1751-1766. doi:10.1007/s00429-015-1001-3

22. Meier EL, Johnson JP, Pan Y, Kiran S. The utility of lesion classification in predicting language and treatment outcomes in chronic stroke-induced aphasia. *Brain Imaging and Behavior*. 2019;13(6):1510-1525. doi:10.1007/s11682-019-00118-3

23. Moulton E, Magno S, Valabregue R, et al. Acute Diffusivity Biomarkers for Prediction of Motor and Language Outcome in Mild-to-Severe Stroke Patients. *Stroke*. 2019;50(8):2050-2056. doi:10.1161/STROKEAHA.119.024946

24. Blom-Smink M, Verly M, Spielmann K, Smits M, Ribbers GM, van de Sandt-Koenderman MWME. Change in Right Inferior Longitudinal Fasciculus Integrity Is Associated With Naming Recovery in Subacute Poststroke Aphasia. *Neurorehabil Neural Repair*. 2020;34(9):784-794. doi:10.1177/1545968320940982

25. Hartwigsen G, Stockert A, Charpentier L, et al. Short-term modulation of the lesioned language network. de Lange FP, Hillis AE, Hillis AE, Den Ouden D, eds. *eLife*. 2020;9:e54277. doi:10.7554/eLife.54277

26. Hula WD, Panesar S, Gravier ML, et al. Structural white matter connectometry of word production in aphasia: an observational study. *Brain*. 2020;143(8):2532-2544. doi:10.1093/brain/awaa193

27. Keser Z, Meier EL, Stockbridge MD, Hillis AE. The role of microstructural integrity of major language pathways in narrative speech in the first year after stroke. *Journal of Stroke and Cerebrovascular Diseases*. 2020;29(9):105078. doi:10.1016/j.jstrokecerebrovasdis.2020.105078

28. Zyryanov A, Malyutina S, Dragoy O. Left frontal aslant tract and lexical selection: Evidence from frontal lobe lesions. *Neuropsychologia*. 2020;147:107385. doi:10.1016/j.neuropsychologia.2020.107385

29. Gajardo-Vidal A, Lorca-Puls DL, team P, et al. Damage to Broca's area does not contribute to long-term speech production outcome after stroke. *Brain*. 2021;144(3):817-832. doi:10.1093/brain/awaa460

30. Kim G, Jeong B, Choi M, Kim WS, Han CE, Paik NJ. Neural substrates of subcortical aphasia in subacute stroke: Voxel-based lesion symptom mapping study. *Journal of the Neurological Sciences*. 2021;420:117266. doi:10.1016/j.jns.2020.117266

31. Kourtidou E, Kasselimis D, Angelopoulou G, et al. The Role of the Right Hemisphere White Matter Tracts in Chronic Aphasic Patients After Damage of the Language Tracts in the Left Hemisphere. *Frontiers in Human Neuroscience*. 2021;15. Accessed March 15, 2022. https://www.frontiersin.org/article/10.3389/fnhum.2021.635750

32. Malherbe C, Cheng B, Königsberg A, et al. Game-theoretical mapping of fundamental brain functions based on lesion deficits in acute stroke. *Brain Communications*. 2021;3(3):fcab204. doi:10.1093/braincomms/fcab204

33. Martinez Oeckel A, Rijntjes M, Glauche V, et al. The extreme capsule and aphasia: proof-of-concept of a new way relating structure to neurological symptoms. *Brain Communications*. 2021;3(2):fcab040. doi:10.1093/braincomms/fcab040

34. Sihvonen AJ, Ripollés P, Leo V, et al. Vocal Music Listening Enhances Poststroke Language Network Reorganization. *eNeuro*. 2021;8(4). doi:10.1523/ENEURO.0158-21.2021

35. Soliman RK, Tax CMW, Abo-Elfetoh N, et al. Effects of tDCS on Language Recovery in Post-Stroke Aphasia: A Pilot Study Investigating Clinical Parameters and White Matter Change with Diffusion Imaging. *Brain Sciences*. 2021;11(10):1277. doi:10.3390/brainsci11101277

36. Zhang B, Chang J, Park J, et al. Uncinate fasciculus and its cortical terminals in aphasia after subcortical stroke: A multi-modal MRI study. *NeuroImage: Clinical*. 2021;30:102597. doi:10.1016/j.nicl.2021.102597

37. Zhang J, Zheng W, Shang D, et al. Fixel-based evidence of microstructural damage in crossing pathways improves language mapping in Post-stroke aphasia. *NeuroImage: Clinical*. 2021;31:102774. doi:10.1016/j.nicl.2021.102774

38. Bae CR, Na Y, Cho M, Hwang YM, Tae WS, Pyun SB. Structural Changes in the Arcuate Fasciculus and Recovery of Post-stroke Aphasia: A 6-Month Follow-up Study using Diffusion Tensor Imaging. *Neurorehabil Neural Repair*. 2022;36(9):633-644. doi:10.1177/15459683221121752

39. Braun EJ, Billot A, Meier EL, et al. White matter microstructural integrity pre- and post-treatment in individuals with chronic post-stroke aphasia. *Brain and Language*. 2022;232:105163. doi:10.1016/j.bandl.2022.105163

40. Sihvonen AJ, Sammler D, Ripollés P, et al. Right ventral stream damage underlies both poststroke aprosodia and amusia. *European Journal of Neurology*. 2022;29(3):873-882. doi:10.1111/ene.15148

41. Zhong AJ, Baldo JV, Dronkers NF, Ivanova MV. The unique role of the frontal aslant tract in speech and language processing. *NeuroImage: Clinical*. 2022;34:103020. doi:10.1016/j.nicl.2022.103020

42. Egorova-Brumley N, Dhollander T, Khan W, Khlif MS, Ebaid D, Brodtmann A. Changes in White Matter Microstructure Over 3 Years in People With and Without Stroke. *Neurology*. 2023;100(16):e1664-e1672. doi:10.1212/WNL.0000000000207065

43. Olivé G, Peñaloza C, Vaquero L, Laine M, Martin N, Rodriguez-Fornells A. The right uncinate fasciculus supports verbal short-term memory in aphasia. *Brain Struct Funct*. 2023;228(3):875-893. doi:10.1007/s00429-023-02628-9

44. Sihvonen AJ, Vadinova V, Garden KL, et al. Right hemispheric structural connectivity and poststroke language recovery. *Human Brain Mapping*. 2023;44(7):2897-2904. doi:10.1002/hbm.26252

45. Soliman RK, Tax CMW, Abo-Elfetoh N, Zaitoun MMA, Khedr EM. Constrained spherical deconvolution -based tractography of major language tracts reveals post-stroke bilateral white matter changes correlated to aphasia. *Magnetic Resonance Imaging*. 2023;95:19-26. doi:10.1016/j.mri.2022.10.004

46. Xiong Y, Khlif MS, Egorova-Brumley N, Brodtmann A, Stark BC. Neural correlates of verbal fluency revealed by longitudinal T1, T2 and FLAIR imaging in stroke. *NeuroImage: Clinical*. 2023;38:103406. doi:10.1016/j.nicl.2023.103406

47. Kümmerer D, Hartwigsen G, Kellmeyer P, et al. Damage to ventral and dorsal language pathways in acute aphasia. *Brain*. 2013;136(2):619-629. doi:10.1093/brain/aws354

48. Bonilha L, Rorden C, Fridriksson J. Assessing the Clinical Effect of Residual Cortical Disconnection After Ischemic Strokes. *Stroke*. 2014;45(4):988-993. doi:10.1161/STROKEAHA.113.004137

49. Kuceyeski A, Navi BB, Kamel H, et al. Exploring the brain's structural connectome: A quantitative stroke lesion-dysfunction mapping study. *Human Brain Mapping*. 2015;36(6):2147-2160. doi:10.1002/hbm.22761

50. Bonilha L, Gleichgerrcht E, Nesland T, Rorden C, Fridriksson J. Success of Anomia Treatment in Aphasia Is Associated With Preserved Architecture of Global and Left Temporal Lobe Structural Networks. *Neurorehabil Neural Repair*. 2016;30(3):266-279. doi:10.1177/1545968315593808

51. Gleichgerrcht E, Fridriksson J, Rorden C, Nesland T, Desai R, Bonilha L. Separate neural systems support representations for actions and objects during narrative speech in post-stroke aphasia. *NeuroImage: Clinical*. 2016;10:140-145. doi:10.1016/j.nicl.2015.11.013

52. Gleichgerrcht E, Kocher M, Nesland T, Rorden C, Fridriksson J, Bonilha L. Preservation of structural brain network hubs is associated with less severe post-stroke aphasia. *Restorative Neurology and Neuroscience*. 2016;34(1):19-28. doi:10.3233/RNN-150511

53. Yourganov G, Fridriksson J, Rorden C, Gleichgerrcht E, Bonilha L. Multivariate Connectome-Based Symptom Mapping in Post-Stroke Patients: Networks Supporting Language and Speech. *J Neurosci*. 2016;36(25):6668-6679. doi:10.1523/JNEUROSCI.4396-15.2016

54. Bonilha L, Hillis AE, Hickok G, den Ouden DB, Rorden C, Fridriksson J. Temporal lobe networks supporting the comprehension of spoken words. *Brain*. 2017;140(9):2370-2380. doi:10.1093/brain/awx169

55. Del Gaizo J, Fridriksson J, Yourganov G, et al. Mapping Language Networks Using the Structural and Dynamic Brain Connectomes. *eNeuro*. 2017;4(5). doi:10.1523/ENEURO.0204-17.2017

56. Marebwa BK, Fridriksson J, Yourganov G, Feenaughty L, Rorden C, Bonilha L. Chronic post-stroke aphasia severity is determined by fragmentation of residual white matter networks. *Sci Rep*. 2017;7(1):8188. doi:10.1038/s41598-017-07607-9

57. Pustina D, Coslett HB, Ungar L, et al. Enhanced estimations of post-stroke aphasia severity using stacked multimodal predictions. *Human Brain Mapping*. 2017;38(11):5603-5615. doi:10.1002/hbm.23752

58. Fridriksson J, den Ouden DB, Hillis AE, et al. Anatomy of aphasia revisited. *Brain*. 2018;141(3):848-862. doi:10.1093/brain/awx363

59. Hope TMH, Leff AP, Price CJ. Predicting language outcomes after stroke: Is structural disconnection a useful predictor? *NeuroImage: Clinical*. 2018;19:22-29. doi:10.1016/j.nicl.2018.03.037

60. Xing S, Mandal A, Lacey EH, Skipper-Kallal LM, Zeng J, Turkeltaub PE. Behavioral Effects of Chronic Gray and White Matter Stroke Lesions in a Functionally Defined Connectome for Naming , Behavioral Effects of

Chronic Gray and White Matter Stroke Lesions in a Functionally Defined Connectome for Naming. *Neurorehabil Neural Repair*. 2018;32(6-7):613-623. doi:10.1177/1545968318780351

61. den Ouden DB, Malyutina S, Basilakos A, et al. Cortical and structural-connectivity damage correlated with impaired syntactic processing in aphasia. *Human Brain Mapping*. 2019;40(7):2153-2173. doi:10.1002/hbm.24514

62. Griffis JC, Metcalf NV, Corbetta M, Shulman GL. Structural Disconnections Explain Brain Network Dysfunction after Stroke. *Cell Reports*. 2019;28(10):2527-2540.e9. doi:10.1016/j.celrep.2019.07.100

63. Baboyan V, Basilakos A, Yourganov G, et al. Isolating the white matter circuitry of the dorsal language stream: Connectome-Symptom Mapping in stroke induced aphasia. *Human Brain Mapping*. 2021;42(17):5689-5702. doi:10.1002/hbm.25647

64. Dickens JV, DeMarco AT, van der Stelt CM, et al. Two types of phonological reading impairment in stroke aphasia. *Brain Communications*. 2021;3(3):fcab194. doi:10.1093/braincomms/fcab194

65. Gleichgerrcht E, Roth R, Fridriksson J, et al. Neural bases of elements of syntax during speech production in patients with aphasia. *Brain and Language*. 2021;222:105025. doi:10.1016/j.bandl.2021.105025

66. Griffis JC, Metcalf NV, Corbetta M, Shulman GL. Lesion Quantification Toolkit: A MATLAB software tool for estimating grey matter damage and white matter disconnections in patients with focal brain lesions. *NeuroImage: Clinical*. 2021;30:102639. doi:10.1016/j.nicl.2021.102639

67. Keator LM, Yourganov G, Basilakos A, et al. Independent contributions of structural and functional connectivity: Evidence from a stroke model. *Network Neuroscience*. 2021;5(4):911-928. doi:10.1162/netn_a_00207

68. Wilmskoetter J, Fridriksson J, Basilakos A, et al. Indirect White Matter Pathways Are Associated With Treated Naming Improvement in Aphasia. *Neurorehabil Neural Repair*. 2021;35(4):346-355. doi:10.1177/1545968321999052

69. Adezati E, Thye M, Edmondson-Stait AJ, Szaflarski JP, Mirman D. Lesion correlates of auditory sentence comprehension deficits in post-stroke aphasia. *Neuroimage: Reports*. 2022;2(1):100076. doi:10.1016/j.ynirp.2021.100076

70. Blackett DS, Varkey J, Wilmskoetter J, et al. Neural network bases of thematic semantic processing in language production. *Cortex*. 2022;156:126-143. doi:10.1016/j.cortex.2022.08.007

71. Erickson BA, Kim B, Deck BL, et al. Preserved anatomical bypasses predict variance in language functions after stroke. *Cortex*. 2022;155:46-61. doi:10.1016/j.cortex.2022.05.023

72. Matchin W, den Ouden DB, Hickok G, Hillis AE, Bonilha L, Fridriksson J. The Wernicke conundrum revisited: evidence from connectome-based lesion-symptom mapping. *Brain*. 2022;145(11):3916-3930. doi:10.1093/brain/awac219

73. McCall JD, Vivian Dickens J, Mandal AS, et al. Structural disconnection of the posterior medial frontal cortex reduces speech error monitoring. *NeuroImage: Clinical*. 2022;33:102934. doi:10.1016/j.nicl.2021.102934

74. Wilmskoetter J, He X, Caciagli L, et al. Language Recovery after Brain Injury: A Structural Network Control Theory Study. *J Neurosci*. 2022;42(4):657-669. doi:10.1523/JNEUROSCI.1096-21.2021

75. Roth R, Busby N, Wilmskoetter J, et al. Diabetes, brain health, and treatment gains in post-stroke aphasia. *Cerebral Cortex*. Published online May 3, 2023:bhad140. doi:10.1093/cercor/bhad140

76. Zevgolatakou E, Thye M, Mirman D. Behavioural and neural structure of fluent speech production deficits in aphasia. *Brain Communications*. Published online October 12, 2022. Accessed November 2, 2022. https://www.research.ed.ac.uk/en/publications/behavioural-and-neural-structure-of-fluent-speech-production-defi

77. Zhao Y, Cox CR, Lambon Ralph MA, Halai AD. Using in vivo functional and structural connectivity to predict chronic stroke aphasia deficits. *Brain*. 2023;146(5):1950-1962. doi:10.1093/brain/awac388

78. Wilmskoetter J, Busby N, He X, et al. Dynamic network properties of the superior temporal gyrus mediate the impact of brain age gap on chronic aphasia severity. *Commun Biol*. 2023;6(1):1-12. doi:10.1038/s42003-023-05119-z

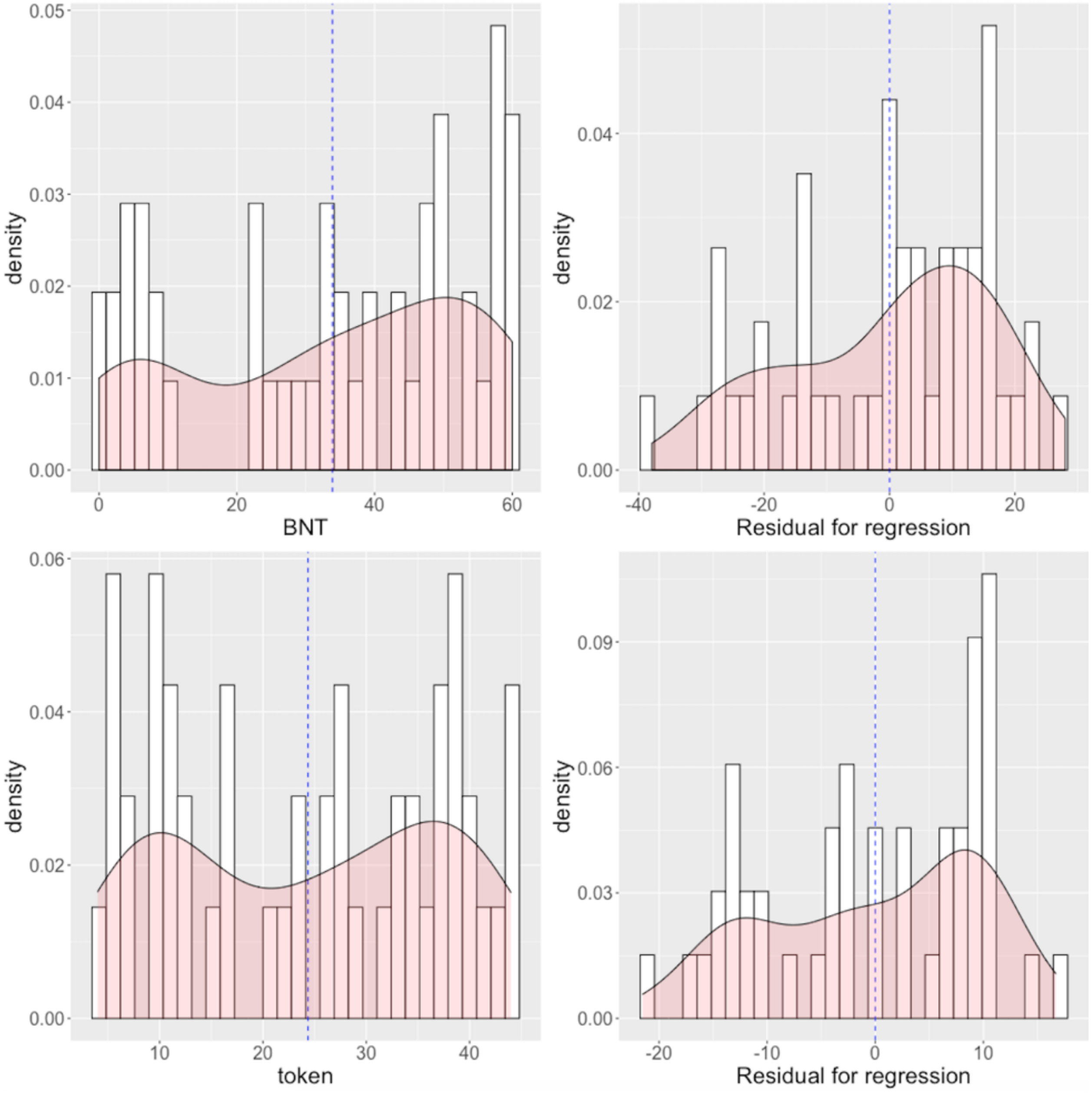

Supplementary Figure 2. The distributions of Boston Naming Test (top) and Token Test (bottom) and their residuals after controlling for lesion volume and scanning protocol (N=50). Blue lines denote the average value.

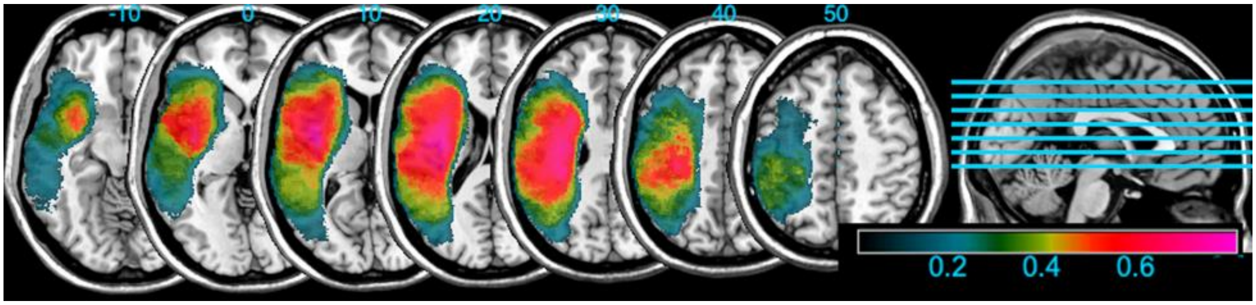

Supplementary Figure 3. The lesion overlap (proportion) of the 50 participants. For the cohort, all the participants' lesion masks were overlapped together. Warmer color indicates a higher damage proportion among the participants.

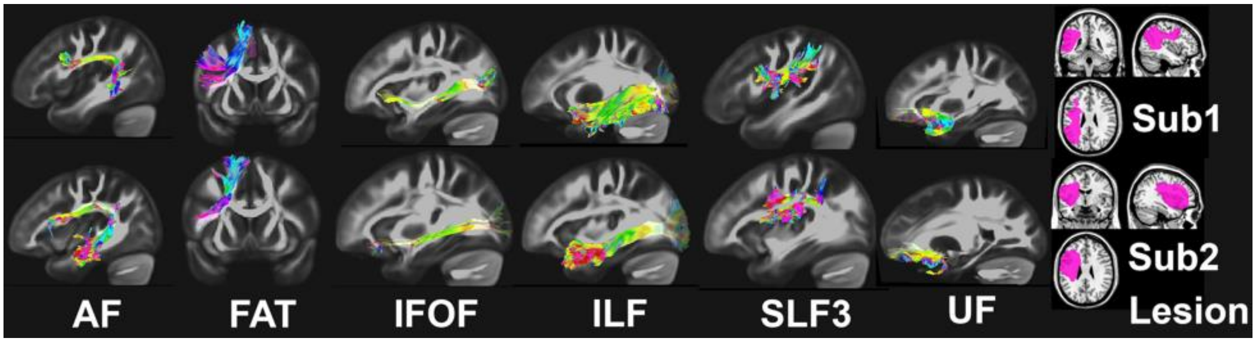

Supplementary Figure 4. The visualization of case 1 and 2's tractography. The automatic tractography was done on DWI images to identify tracts. Two cases tractography results are shown here for the method's evaluation.

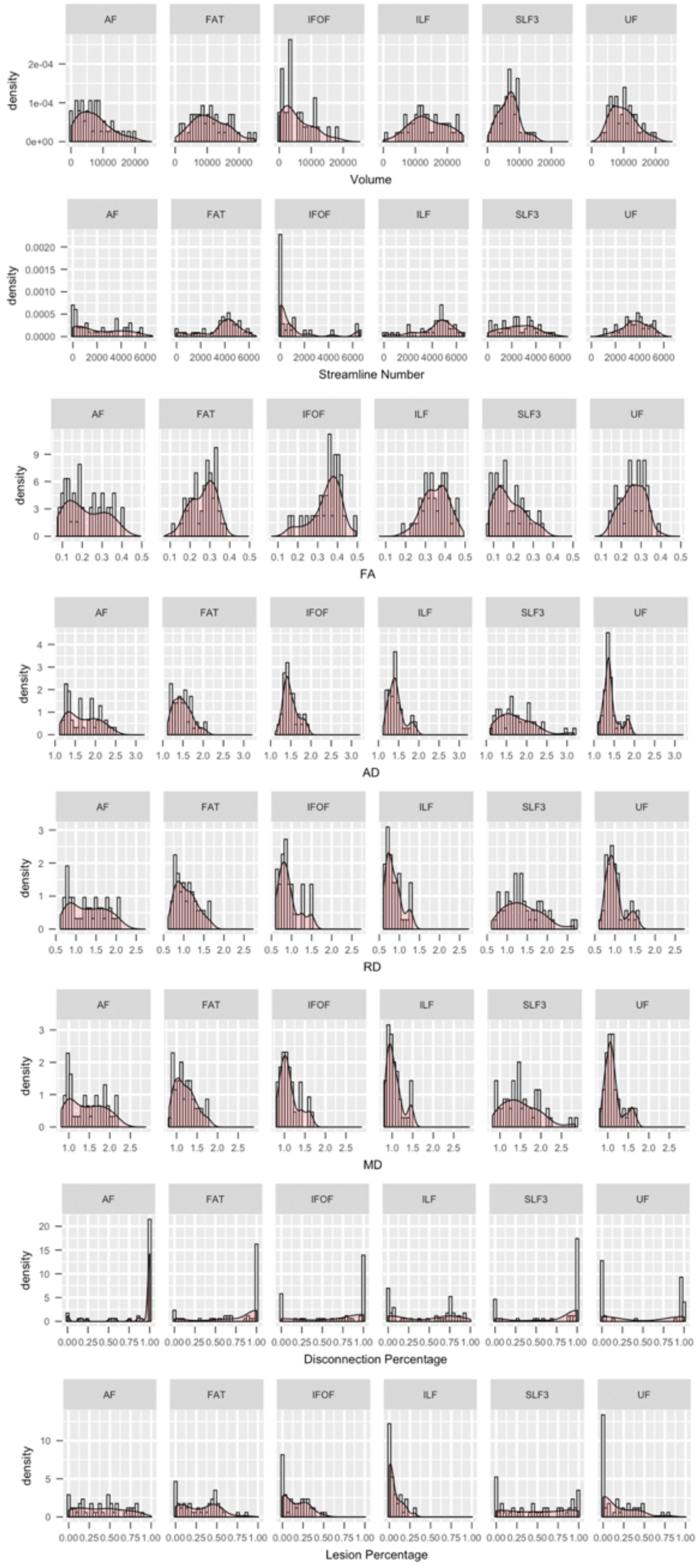

Supplementary Figure 5. The distributions of tract-based metrics (N=50 except for AF [N=43] and IFOF's [N=26] direct metrics). For every tract's metrics, their distribution histograms are shown in detail.

Supplementary Table 3. Definitions and implications of graph theory metrics used in the analysis.

	Input Matrix	Definition	Implication
Characteristic Path Length	Distance	The average shortest path length in the network	A measure of network integration; higher values indicate information must travel through more steps between nodes
Modularity	Connection	Difference of a maximally possible number of within-group connections and a minimally possible number of between-group connections	A measure of network segregation; Higher values indicate the network may be more likely subdivided into clearly groups
Rich Club	Connection	the fraction of connection weights that connect nodes of degree K or higher out of maximum connection weights that nodes might share	A measure of network resilience; Higher values indicate high-degree nodes are more likely mutually interconnected
Strength	Connection	Average of nodes' connection weights in the network	Higher values indicate more connections left in the network
Small-World	Distance and Connection	(Cluster coefficient of the network/random networks) / (characteristic path length of the network / random networks)	A measure of balance of network integration and segregation. Higher values indicate a better balance.
Transitivity	Connection	The ratio of triangles to triplets in the network; Similar to clustering coefficient	A measure of network segregation. High values indicate a densely connected network that is robust to damage

Supplementary Table 4. SCCAN parcel-based CLSM results for naming: CV correlations.

	**Naming** *r* **(CI)**	**Token** *r* **(CI)**
Whole Brain		
Direct	0.19 (-0.09-0.44)	0.14 (-0.14-0.40)
Indirect	0.10 (-0.18-0.37)	0.17 (-0.11-0.43)
Language Network		
Direct	0.18 (-0.10-0.44)	0.16 (-0.12-0.42)
Indirect	0.10 (-0.18-0.37)	0.20 (-0.08-0.45)

Note. CI, Confidence Interval. *$p < 0.05$
